# Rodent maze studies: from following simple rules to complex map learning

**DOI:** 10.1007/s00429-024-02771-x

**Published:** 2024-03-15

**Authors:** Kjell Wijnen, Lisa Genzel, Jacqueline van der Meij

**Affiliations:** Donders Institute for Brain, Cognition and Behaviour, Radboud University, Postbus 9010, 6500 GL Nijmegen, The Netherlands

**Keywords:** Rodents, Maze, Navigation, Learning, Memory

## Abstract

More than 100 years since the first maze designed for rodent research, researchers now have the choice of a variety of mazes that come in many different shapes and sizes. Still old designs get modified and new designs are introduced to fit new research questions. Yet, which maze is the most optimal to use or which training paradigm should be applied, remains up for debate. In this review, we not only provide a historical overview of maze designs and usages in rodent learning and memory research, but also discuss the possible navigational strategies the animals can use to solve each maze. Furthermore, we summarize the different phases of learning that take place when a maze is used as the experimental task. At last, we delve into how training and maze design can affect what the rodents are actually learning in a spatial task.

**Supplementary Information:**

The online version contains supplementary material available at 10.1007/s00429-024-02771-x.

## Introduction

Throughout history, maze(-like) structures have been used to either keep subjects confined (e.g., the labyrinth on the Greek island of Crete to trap the Minotaur) or to provide an intriguing puzzle in which the subject needs to find its way from one or more starting locations to a finish point. Mazes fascinate us and they find continuous use in brain research, eliciting novel insights into spatial learning and other cognitive processes. In this review we will provide a historical overview of different rodent mazes, focussing on their design, and provide a rough outline of principles of learning in mazes. Detailed neuroscientific insights generated from these mazes is beyond the scope of this review and has been summarized by others (please see: O’Keefe 1979, 1993; Eichenbaum et al. 1999; Burgess et al. 2002; Lalonde 2002; Martin and Clark 2007; Euston et al. 2012; Spiers and Gilbert 2015; Farzanfar et al. 2023).

In general, a maze is a collection of paths connected through one or multiple choice points, typically leading to one goal location. The use of mazes in studies of learning and memory has a long history in a variety of animals; though most often rodents, such as rats and mice, are the preferred study subjects. While one of the first reported maze studies was conducted by Lubbock (1884) using ants (see Box 1), the first rodents tested in a maze date back to the beginning of the twentieth century (Small 1901). In the beginning, these experiments were primarily aimed at examining the senses (i.e., vision, smell, touch and kinaesthesis) used by animals in finding their way through the maze (e.g., Watson 1907; Hunter 1929a; McFarlane 1930; Honzik 1936). With the ongoing development of various observational and interventional techniques, the focus of maze experiments from the late twentieth century onwards began to shift to investigate the role the brain plays in spatial learning and memory.

Thus far, two main strategies of (goal-oriented) navigation within mazes have been described, whereby the egocentric strategy is centred on the navigator (e.g., relying upon idiothetic cues and online self-localization during navigation) while the allocentric strategy is based on world-centred representations (e.g., environmental cues and location of the goal) (Klatzky 1998; Samanta et al. 2021). For navigation in a maze task this means that the subject can either solve the task by remembering a series of turns and/or decisions at choice points (i.e., egocentric navigation), or by a map-based approach in which navigation towards a goal is guided by the subjects’ knowledge of the environment (i.e., allocentric navigation). The former strategy, typically expressed in the simplest forms of mazes, can be linked to rule learning (e.g., win-stay or win-shift paradigm in a T-maze (Salvetti et al 2014)); while the latter can be linked to map learning or the use of a schema (i.e., an associative network structure, based on multiple similar experiences though it lacks unit detail and is adaptable) such as, goal-directed behaviour in the watermaze (Morris 1981, 1984) or HexMaze (Alonso et al. 2021), respectively.

Which of the two strategies is used by the study subject is not only dependent on the maze setup but also on the training paradigm and how long animals are trained on the same thing. Nowadays, researchers have a wide assortment of mazes to choose from for their study design, from the simple T-, Y-, and W-maze (also known as E- or M-maze) to more complex water-, multiple alleyway and 3D mazes. Most common alleyway mazes are thought to involve temporal mapping (i.e., alternation of left/right movements) while the more complex mazes involve spatial mapping in which a mental representation of the environment is needed to navigate the maze. In addition, in some of the maze setups, the training paradigm used, can force the study subject to shift its initial allocentric navigation strategy to egocentric navigation. Such a shift can for instance occur due to overtraining of a specific maze task or when a limited number of start locations are used.

The type of maze used in studying spatial learning and memory, whether the animal receives a reward at the goal location and/or punishment for taking the wrong route and which study subject is used, all is highly dependent on the objective of the study (for details on maze configurations and methods used in studies throughout navigational learning and memory research, see supplementary Table 1 and 2). Nonetheless, choosing the correct maze or adapting an existing setup is an important preparatory step and should be guided by the type of learning and memory to be studied. Hence, here we will first provide a historical overview of maze experiments, with a focus on rodent studies, in which we classify the various maze tasks used so far by navigational strategies, difficulty level by means of starting and goal locations, and types of memory used in solving the task. Next, we discuss considerations in training and what animals actually learn in mazes. In doing so, we aim to form a reference framework to guide the choice of maze task for future spatial learning and memory research.

## A short history of maze designs

Mazes used in rodent studies come in various shapes and sizes, in 2D and 3D, in dry form or filled with water, can be used in lit conditions as well as in the dark, and can be used in combination with various observational and interventional techniques. Moreover, new maze designs are still being developed to examine specific behaviours as well as to investigate rodent behaviour in the lab that is more closely related to their natural behaviour. Nonetheless, many of the mazes currently used originate from studies performed in the beginning of the twentieth century or are modifications of earlier developed mazes. Hence, in the following section we will present a historical overview of rodent maze designs used in studies of navigational learning and memory over the past > 100 years*.*

### Late 1800s to beginning of 1900s–first, large mazes for rodents

**Fig. 1:**
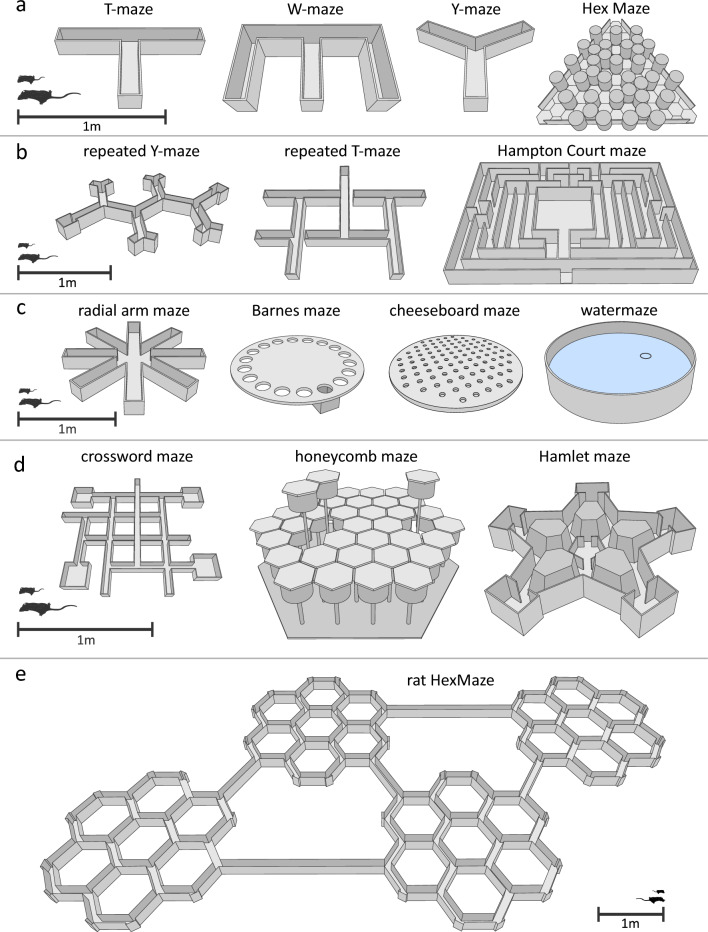
Mazes. Different mazes used for rodent map learning studies. Silhouettes represent relative size of mice and rats compared to mazes. **a** Small, simple maze designs. From left to right: T-maze, W-maze, Y-maze and Hex Maze (Krausz et al. 2023). **b** Mazes containing several binary decision points. From left to right: repeated Y-maze (Warden 1929a, 1929b), repeated T-maze (Tolman and Nyswander 1927) and Hampton Court maze (Small 1901). All three mazes have one dedicated start and goal location. **c** Small, specialized mazes. From left to right: radial arm maze, Barnes maze (Barnes 1979), cheeseboard maze (Dupret et al. 2010) and watermaze (Morris 1981). **d** Configurable (left, middle) and compartmentalized (right) maze designs. From left to right: crossword maze (McNamara et al. 2014), honeycomb maze (Wood et al. 2018) and Hamlet maze (Crouzier et al. 2018). **e** Large scale spatial navigation rat HexMaze (Alonso et al. 2021). All nodes can act as start and goal locations. Maze is completely configurable

Rodents possess the great ability of homing, even in the dark. This behaviour led Edmund Sanford in the late 1800s to suggest to his student, Willard Small, the use of a maze as a resourceful method to study learning in these animals (Logan 2000). Inspired by the maze at the Hampton Court Palace in England, Small (1901) constructed a rodent version to investigate navigational learning and memory in rats (Fig. 1b) (later in modified versions also used by e.g., Carr (1917) and Stone (1928), Supplementary Table 1). First, rats were let into the maze during the night to investigate and familiarize themselves with it. Then, during the day, the rats were placed outside the maze while a food reward was placed inside the maze. The time needed for the rats to find the reward, as well as the number of errors they made (i.e., run into cul-de-sacs or retracing), decreased significantly as the experimental trials progressed. While running several experiments in this maze, Small noticed that two of his rats became blind due to an eye disease. Strikingly, these blind rats performed the experiments as well as the other, non-blind rats and thus did not seem to rely on established memories of visual cues to navigate the maze but possibly made use of other senses such as tactile cues or the memory of body movements.

The role of kinaesthesis (i.e., the perception of ones owns body movements; also called proprioception) and other senses, such as vision, smell and hearing, in maze learning became the main topic of examination in the maze studies following Small’s initial experiments. Using the same maze Watson (1907) tested rats with various impaired senses. First of all, rats that received total visual deprivation, due to removal of their eyeballs, performed similar to non-blinded rats. A similar result was observed when the sense of smell was impaired through removal of the olfactory bulbs. Next, the auditory function was impaired by the annihilation of parts of the middle ear combined with filling the ear with paraffin wax. Notably, the inner ear, and thus the vestibular system, was kept entirely intact. Similarly, no effect on maze performance was observed by these auditory impairments. Equally, removal of all whiskers did not affect the rats’ ability to solve the maze. Lastly, even local anaesthesia of tactical senses did not affect the performance of the rats in the maze. Together, these findings confirmed Small’s idea that outside cues do not play a fundamental role in spatial memory formation. Instead, kinaesthetic information appeared to be among the principal mechanisms of spatial memory (Carr and Watson 1908). Further experiments, using the large-sized Hampton Court maze in its original or altered form, continued the examination of the role different senses (e.g., through removal of whiskers) play in maze learning and memory (Bogardus and Henke 1911; Hicks 1911). 1

While these early studies provided a first insight into navigational learning and memory in rodents, the relatively large maze used only contained one start and one reward location, meaning this maze mainly tested simple path learning or body-turn sequence and thus egocentric navigation in rats.

### Early 1900s to 1940s–the rise of different maze designs

After the initial maze studies, performed with large, two-dimensional mazes with many 90-degrees corners and multiple dead ends (i.e., Hampton Court maze and its altered forms), it did not take long for various other designs to be developed. In 1914, Watson reported on the design of a circular maze setup, combined with the use of a camera lucida, that could be used in its simplest form but could also be adapted to increase complexity. Next, Vincent (1915) introduced an ‘open maze’ which lacks walls that normally would prevent the outlook to neighbouring alleyways (later also used by e.g., Miles (1927); Gilhousen (1931)). Another simple but adaptable maze was described by Dennis (1929), namely the simple rectangular maze. While all of these mazes were novel and adaptable in both size and complexity, they still only tested for path learning and memory used by the rodent in solving the maze. The multiple-choice maze by Burtt (1916), while smaller in size, provided his rats with a maze setup in which they had to learn and memorize specific rules to choose the correct chamber that would lead them to a food reward. Similarly, though less complex in design and thus a conditioned place preference, instead of a navigational task, is the compartment maze used by Helson (1927) in which the rats had to learn that either the dark or the light chamber at the end of the maze would reward them with food.

#### Introduction of the T-maze

The rule learning paradigm in combination with a maze setup is also found back in one of the simplest maze designs, namely where the animal is presented with one binary decision: left or right. Yerkes (1912) published a study in which he demonstrated a T-shaped test apparatus for earthworms (*Allolobophora foetida*) (see Box 1). Shortly afterwards, Hunter (1920), used a T-shaped discrimination box to test his rats on either a simple or a double alternation protocol (Fig. 1a). While his rats were able to master simple alternation (e.g., left, right, left, right, or win-shift), none of the rats were able to do so on the double alternation protocol (e.g., left, left, right, right, or body-turn sequence learning). Next, he examined the same double alternation protocol in an altered form of the maze, named the ‘temporal maze’ (now known as continuous T-maze). Meaning that once the rat had passed through the central alleyway and made a left turn, the chosen arm would lead the rat back to the central alleyway after which the rat needed to decide to either now turn right (i.e., simple alternation) or turn left again (i.e., double alternation). In addition, rats were also trained in a spatial maze, composed of successive T-shaped units (Fig. 1b) (later also used by e.g., Stone and Nyswander (1927)). Together, Hunter’s experiments showed that a rat might learn a spatial maze requiring simple alternation and then run it in terms of kinaesthesis. However, the kinaesthetic cue can only be translated to one behaviour (e.g., always turn right or after turning right, now turn left, a.k.a. win-stay and win-shift; see Sect. “Phases of maze learning”) but not to another (e.g., after twice turn right, now twice turn left, a.k.a. sequence learning). Furthermore, his study shows the need for spatially arranged cues to master a temporal maze and thus path or body-turn sequence learning.

Shortly afterwards, Tolman applied a similar T-maze design to study purpose and cognition in rats (Tolman 1925). This study was the first to report that rats, when placed in a T-maze, base their future decision on their previous choice in that same maze. For example, if the rat decided to go left on the first run, then he would be more likely to go right on the second run. In recurrent runs, rats were found to naturally alternate left and right. This behaviour of alternating between sides is a symptom of the animals’ innate explorative nature. Namely, the most recently visited arm of the maze is best-known and therefore less likely to be explored again (Dember and Fowler 1958). This tendency was later named Spontaneous Alternation Behaviour (SAB; Dennis (1939) also known as win-shift) and is displayed by many other species, including humans (Henderson 1970; Izumi et al. 2013; May and Wellman 2013; Rothacher et al. 2020). To correctly alternate between the two sides, the subject needs to remember which side was previously explored. Therefore, a decrease in SAB could suggest short-term memory impairments (Wenk 2001; Wu et al. 2018).

After these initial studies using the T-maze, multiple variations of this same design followed. Husband (1929) used a repeated T-maze design but slightly adapted the shape of the top part of the T by extending the top of the incorrect arm upwards. This modification of the T-maze, sometimes also called U-maze, makes it more difficult for the rodent to see if the chosen arm leads to the next T-shaped part of the maze (also used by e.g., Dashiell and Bayroff (1931); Snygg (1936)). Next, the Tunnel maze used by Trueblood (1929) which was a triple T-shape made out of glass and placed on a rotational device to study the orientation of rats in a rotated maze. In addition, Hunter (1929a, 1929b) continued his research on alternation behaviour in rats and used the T-maze design to build a three-dimensional maze. In this maze, that consisted of multiple T-shape open alleyways stacked on top of each other, the rats had to follow the path upwards to the reward location by applying the correct alternation rules. A much larger (though two-dimensional), repeated T-maze design was used by Buel (1934), Tolman et al. (1929) built a large, self-recording repeated T-maze and Biel (1940) adapted the T-maze design into a T-shaped water maze to study early-age differences in maze performance. Later, many more renditions of the T-maze (mostly consisting of a multitude of T-shapes in a specific arrangement) would make their appearance in rodent studies (Tolman and Nyswander 1927; Gentry et al. 1948; Schmitzer-Torbert and Redish 2002; Yoder et al. 2011; Olson et al. 2017; Hasz and Redish 2018; Rosenberg et al. 2021; Filatova 2022).

#### Other simple binary mazes

Along with the introduction of the T-maze, another binary decision maze was developed, the Y-maze. The standard Y-maze consists of three identical arms, separated by 120° angles (Fig. 1a). Warden (1929a, 1929b) reported on two different repeated Y-maze designs (also known as Warner-Warden maze) used to study serial learning in rodents (Fig. 1b). In an attempt to standardize the test apparatus and methods, these repeated mazes could be constructed from four modular pieces to make any size of maze. One of the most ingenious elements of this modular design are the (dead) end pieces. Instead of these pieces being similar to normal paths but ending in a wall, they first split into yet two other small corridors. This way, an animal is not able to see if a corridor ends in a dead end before walking into that path. After these initial designs, many more Y-maze designs would follow (Ainge et al. 2007; Bett et al. 2012; Rama et al. 2018; Nagy et al. 2020), sometimes even in combination with the T-maze design (Grieves et al. 2016), as well as other binary designs e.g., U-maze and W-maze (Overton 1964, 1968; Kollner et al. 1988; Frank et al. 2000; Kapellusch et al. 2018) (Fig. 1a).

Both the T- and Y-maze can be utilized for relatively simple tasks such as the SAB (win-shift) test. Nevertheless, although these mazes might look very similar, each serves a specific purpose in memory research. During a Y-maze experiment, an animal could continue its trajectory into one of the arms if it is running alongside a wall. During a T-maze experiment, however, animals would collide with a wall if they use the same tactic. Furthermore, they cannot look inside one of the arms until arrival at the intersection. Thus, in a T-maze the animal must make an active decision between left and right without being able to see what is ahead (Stewart et al. 2011). Nevertheless, the Y-maze also presents several advantages. First of all, the 120° angles (for difference on 90 versus 120 degrees angles see (Coutrot et al. 2022)) make the maze more natural to the animal which results in a shorter learning period (Smit 2022). In addition, and most notably, the Y-maze is most often used for experiments on continuous SAB (Lenck-Santini et al. 2001), meaning that the animal is not removed from the maze after it has visited one arm but instead is able to continue visiting the other arms without interference of an experimenter.

#### Summary

Most of the maze designs discussed in this section aid the examination of rule learning in rodents, though they could also be used to for instance test working memory. When it comes to rule learning in for instance the T-maze (single or repeated), the rodent would be able to solve the task by making just a single body turn. This makes the maze a temporal maze in the sense that the animal runs through the same units of space in successive units of time. Once run, there are no new cues for the rodent to observe. Furthermore, since at least one alleyway is common to at least two different responses (i.e., central alley, turn right and central alley, turn left), the differential cue for decision making cannot come from the pure use of kinaesthesis. The repeated designs, on the other hand, could in addition to rule learning also be used for the examination of path learning. In both the repeated T- and Y-maze the rodent would need to make multiple body turns to go from start to finish. Hence, these types of mazes can be seen as a spatial maze as the animal enters new units of space through time and thus also encounters new stimuli from the environment.

### Mid to end 1900s–Introduction of specialized mazes

While the interest for using mazes in rodent studies seems to have dwindled during this time period, it nonetheless turned out to be a time in which some of the most well-known and still often used mazes were designed. These distinguished mazes are often used to answer specific research questions for which simple binary mazes cannot be used.

#### Sunburst maze

Tolman et al. (1946) introduced the Sunburst maze. This maze consists of 20 arms, projecting from a central round platform, in a pattern resembling sunbeams spreading across the sky. Rats are first trained to walk via a fixed but indirect path leading to the goal box. Next, the test trial is run in the Sunburst maze in which access to the fixed pathway is now blocked. Instead, an array of alternate pathways is now available from the same central hub, of which only one points in the direction of the goal box. Tolman et al. (1946) found that a considerable number of rats tested on the Sunburst maze choose the correct arm that led to the goal box. They thus concluded that the rats can understand the spatial relationship between the start location and the goal, and are thus able to deduce the shortest path (i.e., correct arm) to get there, this is also known as path-integration. Other studies that made use of this or a modified version of the maze were Gentry et al. (1947); Gentry et al. (1948); Kendler and Gasser (1948); Ritchie (1948); Harley (1979).

#### Plus and radial arm maze

All previously discussed mazes contained, in principle, fixed start and/or end points. However, other mazes have been developed in which both these locations are frequently variable. The simplest maze with more than two options would be a plus ( + ) shaped maze (Dirlam 1969). With this maze design, the navigational method of a rodent can be examined or manipulated (Genzel 2020). If the rodent has learned a certain rule, for instance ‘always turn left to find the goal location’, it should not matter where in the Plus maze the animal is placed to obtain the same behavioural outcome during the probe test. When an animal’s navigation is based on its own position and orientation, it will use so-called egocentric spatial memory to solve the task (Johnsen and Rytter 2021). On the other hand, through pre-training to navigate to a fixed goal from start locations in the other three arms, the animal has learned the position and/or orientation of the maze by intra- and/or extra-maze cues (Aleman-Zapata et al. 2022). In that case, it would return to the correct goal arm regardless of the starting position during the probe test. When the animal’s navigation is dependent on environmental stimuli and landmarks, it is expected to use allocentric spatial memory to solve the task. The Plus maze design can of course be expanded to contain more than four arms in total. In 1976, Olton and Samuelson (1976) published a study in which they used a circular maze with 8 arms to study spatial memory in rats (Fig. 1c). This maze design was named the radial arm maze. In the following years, Olton performed several experiments with this maze, leading to the modern ideas regarding spatial reference memory (Olton and Samuelson 1976; Olton et al. 1977; Becker et al. 1980; Meck et al. 1984). Since then, the radial arm maze has been used in numerous studies of spatial memory (Goodale and Dale 1981; Levin 1988; Lichtman 2000; Mizuno et al. 2000; Ranade et al. 2008; Mei et al. 2020). In its most common usage, the radial arm maze is applied to test for allocentric reference memory and working memory. For instance, three arms are baited (same arms for multiple session); first entry into a wrong arm is counted as reference memory error, while re-entry into an arm would count as working memory error. Depending on the research aim, various adaptations of the training and testing protocol are of course possible. While the 6- and 8-arm versions became the standard for mice and rats, respectively, larger radial arm mazes have also been used (Olton et al. 1977; Levin et al. 1997; Hellweg et al. 2006; Nikbakht et al. 2012) as well as other modified versions (e.g., 8-parallel arm maze (Dale 1982), 4-arm radial maze where the arms connect through an octagonal central box (Hulse and O’Leary 1982), 5-arm maze (Durkin et al. 2000), crossmaze including peripheral alleys connecting the ends of the arms to each other (Roberts et al. 2007) and three-dimensional radial maze or radiolarian maze (Wilson et al. 2015)).

#### Barnes maze

The original Barnes maze (Barnes 1979) consists of a circular platform with 18 round holes along the perimeter, of which one leads to an escape chamber (Fig. 1c). The setup of this maze is based on the natural tendency of rodents to choose an enclosed, dark space over open, bright spaces. Hence, when the rodent is placed in the middle of the brightly lit platform it would be reinforced to find and thus learn the location of the escape box i.e., testing allocentric reference memory. The Barnes maze can therefore be used to investigated spatial learning and memory in rodents. However, since the holes of the Barnes Maze are located only along the perimeter of the platform, this maze still lacks spatial continuity and would thus be able to be solved using non-spatial strategies. Hence, several modifications of the maze have been made, for instance by arranging up to 40 holes across the whole platform (Faizi et al. 2012; Illouz et al. 2020). These modified versions of the Barnes maze encourage the rodents to explore the entire platform, thereby eliminating any bias towards the border of the maze as well as the use of non-spatial strategies (Illouz et al. 2020).

#### Watermaze

Although the possible start and end points are increased with the radial arm and Barnes maze, the number of locations is still limited. As an alternative to the radial arm maze, Richard Morris developed the watermaze (also known as ‘Morris watermaze’ MWM, Morris (1981)), which presents virtually unlimited start and goal locations. The main component of the watermaze is a sizeable circular pool filled with water and located somewhere in this pool is a platform which represents the goal location (Fig. 1c). The rodent is lowered into the water at a random or systematic start location (usually E, W, S, N) and swims in an attempt to find the platform. Although rats and mice are natural swimmers, they prefer to rest on the platform. Therefore, this maze has a natural way of reinforcing the animal to go to the goal location (more on different types of reinforcements, see S1. ‘Reinforcements used in maze studies’). Albeit this task can be used to examine either ego- and allocentric navigation, it is predominantly used for the latter examining reference memory but also one-session memory can be tested (Genzel et al. 2017; Samanta et al. 2021). Like all allocentric spatial navigation tasks, it is important to place cues around the maze for the rodent to orient. In addition, it is imperative that the rodent is not able to see the platform while swimming around to test for memory. For this, the water is usually made opaque by adding a small amount of milk (fresh or powdered), non-toxic white paint or latex powder (Morris 1984; Moran et al. 2002; Mifflin et al. 2021). Later, the delayed-match-to-place (DMP) version of the watermaze was developed, during which the platform location is changed each day to enable testing of every-day instead of reference memory (Steele and Morris 1999).

#### Cheeseboard maze

A dry alternative to the watermaze is the cheeseboard maze (Gilbert et al. 1998; Gilbert and Kesner 2002, 2003; Dupret et al. 2010). This maze is made up of a circular board with many wells, which could all act as a possible goal location containing a food reward (Fig. 1c). The advantage of this maze over the watermaze is the exclusion of water, making it more suitable to use in combination with fragile electronic components (e.g., electrophysiological implant). In addition, being placed in water can be stressful for animals. Therefore, the cheeseboard maze provides a less stressful, cost effective alternative to the watermaze that delivers similar results (Lopez et al. 2010). However, in contrast to the watermaze, usually many trials are performed in each session and only one starting location (i.e., start box) is used, which leads to stereotypical paths between the different goal locations and egocentric coding that develops over the trials. How quickly this develops depends on how experienced the animal is with the maze.

An adaptation of the cheeseboard maze, is the alternating home-random task in a square open field first presented by Pfeiffer and Foster (Pfeiffer and Foster 2013). In this variation a daily-changing home location reward alternates each trial with a random reward in the open field, allowing the capture of path calculation processes from random to home location. However, despite the novel home location each day, with training this task also becomes automatic and hippocampus-independent within a few trials each day (Duszkiewicz et al. 2023).

#### Summary

The mazes presented in this section all have the potential to use more than one start and/or goal location, hence aiding the examination of both egocentric and allocentric memory. The choice of which maze best to use depends on the research question under investigation. For instance, the Sunburst maze in its design is generally used to test for path-integration while the different radial arm mazes are often used to examine allocentric reference memory and working memory in both rats and mice. Though, which memory the animal actually uses to solve the specific maze task, depends on the training paradigm being followed.

### 2000s onwards–Technical development opens a new era of maze designs

With the turn of the century came also a new interest in the use of mazes in studies of spatial navigation, learning and memory. Moreover, technical developments did not only inspire the design of new mazes but also aided in combining maze experiments with both novel recording and interventional techniques (see S2. ‘The use of mazes in combination with observational and interventional techniques’). During this period, various mazes discussed earlier in this review found their way back into the lab, however in this section we will focus on the newly designed mazes and their features.

#### Star water- and Hhamlet maze

Both the star watermaze (Rondi-Reig et al. 2006; Fouquet et al. 2013) and Hamlet maze (Crouzier et al. 2018) are star-shaped and used to study different navigational strategies in rodents and thus simple body turn, body-turn sequence or location memory. Both mazes consist of five central alleys forming a pentagonal ring in the middle of the maze and five peripheral alleys radiating from this central ring. In the Hamlet maze, however, the peripheral alleys split up in two arms, each leading to a different goal box (each containing a different reward) (Fig. 1d). Where the Hamlet maze is a so-called dry maze, the starmaze is in design meant to be filled with water but can be used without as well.

#### Crossword and lattice maze

The earlier described circular shaped mazes have the benefit that both start and goal location are highly adaptable. However, they are not very true to nature, especially when compared to the repeated binary mazes. Instead of consisting of connected corridors that simulate burrows, they are large open spaces. The crossword maze (McNamara et al. 2014) is an intermediate maze design that not only takes aspects from repeated T-mazes but also includes the ability to change start and goal locations in addition to providing the animal with more than one passable route to reach the goal location. These adaptations make the maze suitable for both ego- and allocentric spatial navigation tasks. The crossword maze consists of four boxes, connected by corridors that are assembled in a ‘city block’ manner (Fig. 1d). The start and goal location can be interchanged between these four boxes. In addition, blockades can be placed or removed between each corridor. The placement or removal of blockades is an excellent method to test the flexibility of rodent’ spatial behaviour. The lattice maze, while slightly different in design, follows the same principles as the crossword maze and can thus also be used to test how rodents utilize their internal representation of a spatial structure (i.e., cognitive map) to respond to a change in a learned environment (Okaichi 1996). The triangular shaped Hex Maze (Krausz et al. 2023), while similar in setup (i.e., up to three start/end locations and possibility to place barriers), is relatively small compared to other complex maze designed for rats (Fig. 1a). In each case animals can learn about the fixed environment and use this information to each day learn the current path(s) or body-turn sequence(s) and thus egocentric memory.

#### Honeycomb and Tartarus maze

Both the honeycomb maze (Wood et al. 2018) and the Tartarus maze (de Cothi et al. 2022) are a configurable, open-field maze to test spatial navigation behaviour in rodents. The honeycomb maze consists of 37 hexagon-shaped platforms that can be raised or lowered independently (Fig. 1d), while the Tartarus maze consists of 10 x 10 square units each of which can be removed from the maze to create different maze configurations with gaps between traversable surfaces. Seemingly both mazes are very similar in setup, though the difference lays in the fact that the configuration of the honeycomb maze depends on the choices the rat makes while traversing the maze, while the configuration of the Tartarus maze is predetermined by the researcher. Nonetheless, both mazes have the advantage that both start and goal location, as well as the path taken between them, is highly variable. In each case, the maze is used to test for allocentric, reference memory with an emphasis on testing for path calculation to the goal.

#### Event Arena

In 2001, Richard Morris invented the Event Arena—a 1.5 × 1.5 m open field where “events” take place (Tse et al. 2022). The open field consist of 7 × 7 possible locations of liquid-reward wells (similar to cheeseboard maze) or sandwells. This maze has been used to test for paired-associates learning (Day et al. 2003), schema (Tse et al. 2007), every-day memory (Wang et al. 2010; Takeuchi et al. 2016), and path calculation (Duszkiewicz et al. 2023), in each case depending on the training paradigm. With four possible start locations, this maze usually tests for allocentric orientation, but this also depends on the training paradigm (Broadbent et al. 2020).

#### HexMaze

The earlier described crossword maze presents allocentric spatial navigation research with an apparatus which alleyway design mimics the natural gangways of a rodent’s natural environment. However, the options for start and goal locations, though variable, are still limited. On the other hand, the watermaze presents numerous locations but lacks in size and is less naturalistic. In 2021, Alonso et al. published a study that used a novel spatial navigation task that combined the strengths of both these mazes. This maze, the HexMaze, is a large-scale navigation task for mice, consisting of 30 corridors and 24 crossings. These crossings, from now on referred to as nodes, are all possible start or goal locations. Like with repeated Y-mazes, the corridors of this maze are separated by 120° angles, making the turns more natural but navigation more complex. Similar to how old inner cities in Europe are more difficult to navigate without an internal map than 90° cities like New York (Coutrot et al. 2022). Moreover, the biggest advantage of these angled corridors compared to the ‘block’ structure of the crossword maze, is that animals can only look one node ahead. This prevents the animal from seeing the reward at the goal location from afar and simply walking towards it by sight, circumventing the need to form a cognitive map. In addition, the same trick was used as with the Warner-Warden maze. The edges of the maze are not simply flat walls, but first split into a small corridor before presenting a dead end. Therefore, the animal is not able to know where in the maze it is by simply looking at the node in front, because all look the same. Thus, the animal must rely on forming a cognitive map of the maze, based on the numerous intra- and extra-maze 2D/3D cues (e.g., patterns on the walls and various sized objects spread in and around the maze). With this maze, there are countless possible paths from the start positions to the goal location without real dead ends. Therefore, relative path length is used to measure performance in this maze. The same lab has developed an even larger spatial navigational task for rats rather than mice. The rat HexMaze consist of four times the original HexMaze, connected through two large and three small bridges, spanning 9 × 5 m in size (unpublished) (Fig. 1e). Like the mouse version, each node (96 in total) can serve both as a start as well as a goal location. Both the more naturalistic shape and size of these two mazes as well as the large variability of start and goal locations, make the HexMaze a suitable spatial navigational task to examine allocentric navigation, map learning and spatial memory.

### Summary

From the large Hampton Court maze to smaller binary mazes and back to large-scale mazes like the HexMaze. From walled alleyway mazes that prevent a rodent to look too far ahead while navigation from a fixed starting point to a fixed goal location, to open-field and highly adaptable mazes that challenge the rodent with multiple starting and/or goal locations reachable via a variety of paths. Throughout the past > 100 years many different mazes have been developed to use in rodent navigational studies. Especially the past 20 years saw a surge in new maze designs, even though many earlier designs, albeit often in a modified form, are also still used. Interestingly, new maze design seems to return to the original thought of using large setups and thus aim at representing a more complex, naturalistic testing setup in the laboratory environment. Adaptability of the maze setup as well as the potential use of the maze with different animals along with various observational and interventional techniques, are the key features of the latest maze designs. For future applications of mazes in navigational learning and memory research, a consideration needs to be what the animal is actually learning in each maze and how one can shape the animal’s behaviour to engage the cognitive processes one aims at investigating.

## Phases of maze learning

When animals are exposed to and then trained in a maze to express the desired behaviour, different stages will be followed depending on the type of experiment and maze. Many of the early stages are not well described in articles despite the big impact they can have on output as well as time needed to train.

### Handling

Before animals are placed in mazes, they should be handled to habituate themselves to the experimenter as well as the situation (i.e., being taken out of the home cage and/or being brought to another room). The more time spend on this, as well as how active the play with the animal is, will influence later behaviour in the maze. Well-handled animals are, for instance, more confident when exploring novel environments or very open spaces.

### Habituation, shaping and pre-training

Once the animals are well-handled, a period is usually needed during which the animals are initially exposed and habituated to the training environment, and perhaps specific training is applied for animals to learn behaviours needed in the maze (e.g., digging (Broadbent et al. 2020; Tse et al. 2022)).

The term habituation is commonly used when animals are exposed to the later training environment but no specific training to the goal location is performed. This can range from spending time in the maze with no reward to spending time in the maze with many rewards sprinkled everywhere to enhance exploration tendencies. The main aim of this is to decrease fear and encourage early reward consumption on the first training trials, since animals will not eat until they feel safe. Naturalistic and simple mazes and tasks, often do not need more before experimenters can start their training (Genzel et al. 2019; Alonso et al. 2021).

Some training paradigms will next have a shaping period, during which specific behaviours are taught. For example, in tasks involving digging, the process of digging for reward needs to be taught to the animal. This can take up to a week during which rewards are initially placed on top of sandwells and later submerged deeper and deeper. For the watermaze, shaping can involve the usage of a cued paradigm, where curtains occlude any orienting cues but an object is placed as a cue on top of the submerged platform (or the platform can also be raised to be visible). These trials teach the animals to not swim at the walls but go towards the middle of the pool to find a platform and to not be scared since they will always be saved (i.e., after 60 s animals are guided to the platform).

Shaping is also important during the main training period, to ensure the animal produces the desired behaviour or to inhibit the development of automatic and stereotypical behaviours. For example, if we want to test for long-term memory and the return to the goal location during the subsequent training session(s), it needs to be reinforced that the animal can find food at the old location in general during training. The usage of probe trials (i.e., no food or platform or other motivation present) to test for memory without confounding factors or changing repeatedly the goal location (i.e., to control time exposed to the new information) can lead to extinction of the returning behaviour; thus, such trials should be interleaved with retraining trials—food is normally placed at the old goal location even at the first trial of the day—to shape the behaviour. On the other hand, keeping the start and/or goal location steady for too long, can also lead to habit development (see Sect. “Training–late learning (overtraining)”) and thus switching these locations can also be done to positively shape behaviour.

Shaping in addition has to be species-specific e.g., mice usually need more time to both habituate and shape. Furthermore, the environment itself needs to be adapted to shape specific behaviours and this needs to be done differently in each species. For example, to induce pause-thinking/deliberation moments (i.e., instances where the animal stops moving around the environment, most often seen at choice points, to look around and potentially plan/decided the “correct” route to take) that engage the explicit memory system, choice points are sufficient for rats, while these should be in addition dark and safe for the same behaviour to be observed in mice. Mice will further prefer tighter and more enclosed environments. Choice of orienting cues will also shape strategy, meaning that very dominant cues close to the goal can create beaconing strategies while distal, overlapping 3D cues could help with allocentric orientation, though the animal’s vision-capabilities should be considered (Genzel 2021). Albino strains are practically blind and even pigmented rats have a vision of -1.5.

After habituation and shaping, pre-training will be applied for specific tasks. Pre-training usually means an animal is taught a certain rule that allows us to have a behavioural read-out of the cognitive process targeted. However, what is considered pre-training for one study, may be the key focus of another.

### Training–rule learning

Each type of training will come with certain rules that the animals have to learn to receive a reward in the task. It can be a simple, natural rule (e.g., if you found food/safety at a certain location, go back to that location to find it again), which tends to need no pre-training. Animals will often on the second trial go to the previous goal location, which then already reinforces the return behaviour as a future strategy. Also, tasks involving exploitation of natural behaviours such as novelty-seeking and curiosity (e.g., object exploration tasks or general maze exploration experiments) tend to need little to no pre-training.

Then there are some tasks that use natural tendencies of animals but need pre-training to induce a group behaviour and/or suppress the natural occurrence of variability in behavioural output. For example, animals will naturally show both win-shift (e.g., if food found on left arm of T-maze, go next time right) as well as win-stay (e.g., if food found on left arm, return to that arm) behaviours since in natural environments both can—depending on the situation—lead to continuous reward. You should keep returning to the apple tree but once you have collected the individually (fallen) fruits you should shift to a new potential location (see discussion on SAB behaviour in Sect. “Introduction of the T-maze”). Some animals will prefer one strategy, while others will prefer the other strategy and in each case all animals will still spontaneously exhibit both when tested multiple times (Salvetti et al. 2014). However, for practical reasons one rule can be reinforced and thus all animals should perform it most of the time (with shifts of strategy or rule either deliberately manipulated or scored as error trials). This basic rule-training is needed for most tasks involving T-maze, W-maze or other alternation tasks.

### Training–early learning

The early training period is usually the time point when the animal is really doing what most researchers want them to do. The task is novel to them and they are engaged, and will use e.g., short-term memory in most usages of T-, Y- and W-mazes or slowly build up a long-term/reference memory in tasks involving fixed goal locations such as the watermaze, Barnes maze and any other larger maze. Yet, the animal will still be prone to making errors as part of the learning process. Early learning is likely to still involve what would be the explicit memory system in humans, primarily involving the hippocampus and cortical areas.

### Training–late learning (overtraining)

After longer periods of training, animals will start to shift to the most efficient strategy. When this occurs and what the subsequent strategy is, will depend on the difficulty of the trained content as well as any behavioural shaping that is applied. In simple T-, Y- and W-mazes animals may shift quickly (within a few weeks) to automatized behaviours and habits, that likely do not involve the hippocampus but instead rely on other brain regions such as the striatum.

In larger environments, this process may take longer but will also occur, especially if the respective behaviours are very repetitive due to a restricted number of start and/or goal locations (i.e., resulting in restricted possible paths and body-turn sequences). In each case, whatever is fixed for a long time, will be harder to change later. For example, in reference memory tasks, during which the goal location is kept the same over many weeks, learning a new goal location—reversal learning—tends to take multiple trials and tests for cognitive flexibility. Returning to the specific goal becomes a habit that is difficult to break.

In contrast, some tasks involve daily switching of goal locations, such as the cheeseboard maze, the event arena or the DMP version of the watermaze, which encourages the use of short-term memory for longer than in simple T-, Y- or W-mazes. However, often in the end this leads to strategies with stereotypical paths that are often egocentric based. Nonetheless, this can be counteracted with specific shaping practices such as irregular and non-daily switching of goal locations to encourage long-term memory usage and enforcing different routes via varying start locations (Broadbent et al. 2020).

The key element of late learning, is that while the behavioural output may be similar to or better than early learning, cognitive strategies will start to differ if explicit shaping is not applied (and sometimes despite that). This stage often comes with near perfect performance, which is often desired by the experimenter. However, this also means that the behaviour is likely overtrained and has become an automatic, stereotypical response relying on subcortical brain areas.

If, however, very complex environments are combined with flexible use of many start and goal locations in addition to specific shaping of behaviour (such as not too many trials per day and not training for too many days in a row), the animal can remain engaged using the explicit memory system and would show pause-thinking moments even late in training (Tse et al. 2007; Alonso et al. 2021). This allows testing of cognitive processes that are only present late in training such as schema effects.

## Summary

Every behavioural experiment will need some form of habituation, shaping and/or training phase, what exactly and how much is determined by the cognitive process to be investigated. The key for experimenters is to keep in mind that the same behavioural output can be achieved by a variety of strategies and thus underlying neuronal circuits. Targeting the right time point or shaping the behaviour to keep the animal with the target cognitive strategy, is critical for good experiments. Animals will always strive to the “laziest” or use the most economic strategy, which often leads to habit and skill behaviour not involving the explicit memory system most researchers aim to investigate.

## What the animal actually learns?

There is a large variety of cognitive processes that can be tested in mazes. The same maze can be used for different research questions and often the same experiments and behaviours can be analysed in respect to different cognitive processes contributing to the overall behavioural outcome. Many of such we have already covered in the section above e.g., when researchers want to investigate how animals learn rules, they will focus on early training of such a rule. We also discussed how the longer duration the animal spends in the same task can lead to shifting strategies, which in turn can affect if the animal is exhibiting the desired cognitive process. What we want to focus on next, is how training and maze design can affect what exactly the animal is learning in spatial mazes.

### Simple rule/body turn

**Fig. 2:**
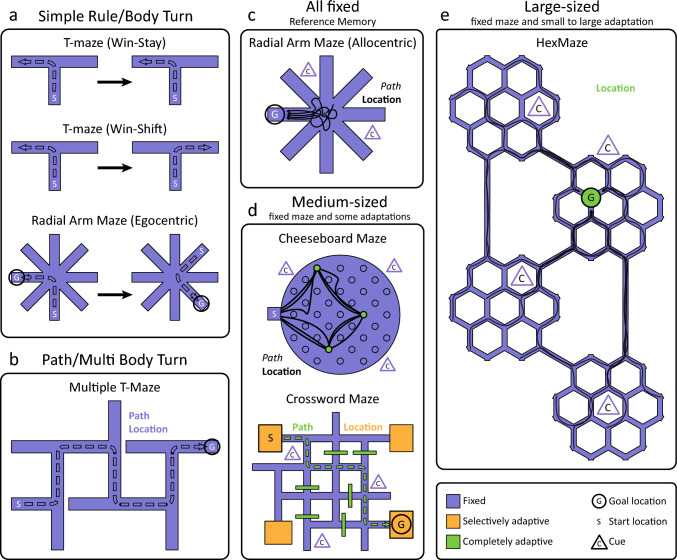
What they learn. What an animal learns is determined by the maze design. **a** Simple mazes can be used to learn a simple rule (e.g., win-stay or win-shift). For this, the maze is fixed and the learning process is independent of the environment. **b** Complex mazes with one path from the start location to the goal are used to learn paths, or a sequence of body turns. The animal only learns how to reach the goal from one start point and learning is independent of cues. **c** When start location and/or orientation are not fixed, the animal will rely on its environment to learn a spatial location. Black lines indicate possible trial runs; the animal is continuously placed in the middle with different orientations. The animal uses intra- and extra-maze cues for this allocentric strategy. Overtraining can lead to possible path learning. **d** Mazes with selectively adaptive start and goal locations. For the cheeseboard maze, animals will find a reward at three wells in the maze (Dupret et al. 2010). These locations are fixed per training but are completely adaptive between training sessions. Black lines indicate possible trial runs; the animal starts in the start box (indicated ‘S’) and then visits all three wells in order, before returning to the start box to start over. Overtraining can lead to possible path learning. The crossword maze contains four possible start and goal locations that can be interchanged between training sessions. Paths between these locations are completely adaptive through the use of barriers (McNamara et al. 2014). **e** A large-sized maze with completely adaptive goal and start locations. Per training session, one goal location is chosen after which the animal will consecutively be placed at random start locations. With the use of environmental cues, the animal will find a way to the goal location (indicated by the black lines)

Simple mazes such as T-, Y-, W-, and plus-maze but also more complex such as radial-arm and starmaze (Rondi-Reig et al. 2006) are often used to train a simple rule such as win-shift or win-stay and then test for task-engagement and short-term memory (but see Sect. “Training–Rule learning” and “Training–late learning (overtraining)”) (Fig. 2a). They can also be used to train simple body movements such as a left-turn—a classic form of egocentric spatial memory dependent on the striatum. In these types of tasks, animals need to learn a fixed spatial environment combined with a simple behaviour. Thus, these tasks will often be learned quite fast and animals will also quickly become overtrained.

### Path/multi body turn

With more complex mazes, especially classic mazes such as the Hampton Court maze, often a fixed start and goal location is used (Fig. 2b). This leads to animals learning a fixed path through a fixed environment, thus testing egocentric navigation. While in this case it will take longer for the animal to learn and perform perfectly in comparison to a simple maze, the basic route even in very large mazes is often not too complex; thus, this type of learning can also quickly become overtrained and automatic, involving less of the explicit memory system over time. Interestingly, such longer series of body turns can, due to the sequence component, still involve hippocampal processing (Cabral et al. 2014).

One main caveat often present in tasks with larger mazes, is the absence of cleaning between trials. If the start and goal locations are fixed, the animals are likely to leave smell traces that will lead to a memory-independent navigational strategy. The same issue arises when the maze is not cleaned between animals and the same goal location is used. Subsequent animals can use the smell traces left by others. Furthermore, if enough trials are performed, a general smell gradient to the goal can be created even despite the usage of different starting locations and thus different paths.

### All fixed–reference memory

Many classic mazes such as the watermaze, Barnes maze and radial arm maze will involve one fixed goal location in a fixed spatial environment but many changing start locations (Fig. 2c). Thus, the training procedure leads to shaping and learning of a spatial location as reference memory (allocentric strategy) instead of a path. However, the existence of multiple starting points is not the only procedure to reliably ensure that an animal learns via an allocentric learning strategy. It is possible that animals solve these mazes based on navigating towards an individual extra-maze cue—known as beaconing (Collett et al. 1986; Pearce et al. 1998). Researchers need to keep this in mind and use correct controls when performing experiments where reference memory is tested. Ideally goal locations should not be placed too close to one very salient cue, instead locations should be chosen that can only be identified by recognizing multiple cues and their relation to each other. Further, experimenters can check if animals approach the goal from the same location in the maze, independent where the start location was. This is an indication that beaconing or egocentric strategies are used, despite allocentric training procedures.

Reference memory mazes are usually medium sized (1–2 m) and the changing start location forces the animal to stay engaged in the environment to orient itself in relation to the goal; thus, here it takes longer for overtraining to occur and the explicit memory system is engaged over longer training periods. However, also in these tasks, returning to the goal location will become at one point a habit as seen in the difficulty of reversal learning. How long it takes to develop into a habit, will depend on the amount of different start locations that are possible and thus how varied the trials can be.

### Medium-sized, fixed maze and some adaptation

More recently, medium-sized mazes that remain fixed throughout training are frequently combined with components that switch from day to day (Fig. 2d). Often in these cases animals are pre-trained on the rule (e.g., there will be new goal locations or other maze changes each day but once you find it, it will be stable for the rest of the day) until they reach a target performance level. The experimental period begins thereafter, and performance depends on the acquisition of today’s new information, modelling every-day memory (e.g., where did I park my car?). In tasks such as the DMP watermaze, event arena or cheeseboard maze, animals should be learning only a new location, thus engaging the hippocampus. However, depending on the number of trials used each day and the number of possible start locations for these different trials, also here returning to today’s goal location can become a simple skill or habit if the animals are trained for a very long time (weeks/months).

Another variation of the same principle is the crossword maze, where the maze with cues is fixed and the start and goal locations are semi-variable (four locations of which two are used each day, one for start and one for goal), but the possible inclusion of walls obstructing passages, leads to animals learning one fixed path per day and thus multi-body-turn sequence or egocentric memory. The maze itself is due to the 90-degree angles simple (see Sect. “Other simple binary mazes” and “HexMaze”), however, the complexity of the possible maze configuration keeps the animals and their explicit system longer engaged. Nonetheless, even with this more varied system, at one point the overlap between the daily path and previously used paths becomes very large with the corresponding risk of automatization and skill development.

In all these types of tasks, animals will initially use short-term memory to solve the every-day memory paradigm and can use the hippocampus for the multi-body-turn sequence (Cabral et al. 2014). However, due to the increasing load of interference of each day’s “every-day-memory” on the subsequent day’s learning, animals are likely to develop alternative strategies and thus develop a skill in solving the task^2^ (Duszkiewicz et al. 2023).

### Large-sized, fixed maze and small to large adaptation

A final variant are large mazes that can have both small adaptations such as changing the goal location as well as larger adaptations such as the inclusion of walls or restructuring the whole maze layout (Fig. 2e). Also in such mazes, the number of possible start and goal locations used will influence navigational strategies employed by the animals. For ideal engagement of the explicit memory system–as often can be recognized due to so-called VTE behaviour (vicarious trial and error i.e., pause-and-look behaviour often seen at choice points (Redish 2016))—start locations should be randomly selectable from the whole maze and changed from trial to trial. Goal locations should remain stable for multiple days/weeks to enable behavioural shaping (i.e., it is worth to return to the previous trained location) and the expression of long-term memory, but should be switched often enough to not induce a fixed and less-flexible reference memory with resulting habit behaviour. This allows stable testing of the same cognitive strategy for longer time periods and also enables access to cognitive processes that are dependent on extensive previous knowledge such as schemas.

## Summary

With different mazes and training procedures, animals will learn and express different aspects of spatial memory. What all have usually in common, is the fixed environment and spatial cue configuration that is often already “learned” during habituation, but will be refined with each exposure to the maze. This spatial environment is the scaffold used for any memory thereafter, which can be simple body-turns or rules as well as paths and locations. However long individual start and goal locations are used in training, will determine if the animal learns a path or location, and how long it takes for the behaviour to be overtrained and become a habit or skill.

## Conclusion

In this article we provided an overview of simple and complex mazes used in behavioural and neuroscience research. Diversity of structure and training paradigm is mapped onto the diversity of research questions posed. We explored what is actually learned in each maze and discussed how training boundaries, such as number of trials and length of training, can influence which strategy the animal may employ. We summarize, that there is no optimal maze or paradigm, instead mazes should be chosen by the type of cognition to be queried. Nevertheless, the experimenter should also remain alert to how the chosen training regime (such as daily versus spaced training, continuous reward locations versus shifted ones) will influence how fast habits instead of explicit memory use will develop.

### Supplementary Information

Below is the link to the electronic supplementary material.

Supplementary file1 (PDF 377 KB)

## Data Availability

There is no data associated to this publication.

## Box 1: Mazes and species other than rodents

This review focused on different types of mazes used in rodent research, in which rats and mice form the majority of subjects used. There are indeed a number of advances in using rodents over other animal species when deploying a maze as the study apparatus. First of all, in their natural habitat, rodents live in burrows which do not only form their homebase to return to after foraging but also in and of itself are a winding network of gangways. Besides the burrow network, especially rats and mice, also navigate along stereotypical paths—rat highways—leaving or returning to the home while foraging for food. Second, for testing in lab conditions, rodents are relatively easy to keep due to their size, dietary needs and housing conditions. At last, unlike research in humans, research in rodents provides the possibility to combine examination of maze navigational behaviour with various interventional and recording techniques.

While rodents seem to be the preferred model, the use of mazes in research is definitely not restricted to these study subjects. Already during the end of the nineteenth century, Lubbock (1884) used a maze-like structure to study navigational information transfer between ants. Since then, a variety of insect species e.g., bumblebees (Giurfa et al. 2001; Mirwan and Kevan 2015), honeybees (Zhang et al. 1996, 2006; Pahl et al. 2007), wasps (Moreyra and Lozada 2021), woodlice (Hughes 1967), ants (Harrison et al. 1989; Jander 1990; Czaczkes and Ratnieks 2012; Foster et al. 2014; Czaczkes and Heinze 2015; Czaczkes et al. 2016; Saar et al. 2017), and crickets (Wessnitzer et al. 2008), have been studied in various types of maze tasks. Furthermore, from the late twentieth century onwards there is a large increase in the use of diverse study subjects in maze research, ranging from different species of fish and crustaceans (e.g., crawfish (Yerkes and Huggins 1903); paradise fish (Warren 1960); zebra fish (Darland and Dowling 2001); cuttlefish (Cartron et al. 2012); European shore crab (Davies et al. 2019) and gilthead seabream (Arechavala-Lopez et al. 2020)) and reptiles (e.g., turtles (López et al. 2000); tortoises (Mueller-Paul et al. 2012a, 2012b); jewelled lizards (Mueller-Paul et al. 2012a, 2012b) and velvet geckos (Abayarathna and Webb 2020)) to birds (e.g., English house sparrow (Porter 1906); domestic chick (Jones et al. 1999) and pigeons (Roberts and Van Veldhuizen 1985)) and mammals (e.g., raccoon (Hunter 1928); pig (Jansen et al. 2009; Kornum and Knudsen 2011); dog (Salvin et al. 2011); rhesus monkey (Kinnaman 1902; Wang et al. 2007); bats (Ruczynski and Siemers 2011), and humans (Iglói et al. 2009; Rothacher et al. 2020)). Moreover, even amoeboid organisms like slime mould (Nakagaki et al. 2000), nematodes like *C. elegans* (Qin and Wheeler 2007; Gourgou et al. 2021) and annelids like earthworms (Datta 1962) have been examined in a maze setting.

## Footnotes

1 For a wonderful layman’s approach to mouse maze see https://woodgears.ca/farm/mouse_maze.html.

2 Of note, we notice the same evolution of strategies in our students when they learn how to train animals. Initially, all is new and they need a lot of cognitive resources to perform the training correctly. Then the period (usually 4–8 weeks into the internship) of increased interference of the previous trial's start and goal locations makes them have more mistakes (e.g., reward placed wrong or animal placed wrong). Finally, after 2–3 months they are experts, training has become a habit/skill and they do not make mistakes despite listening to podcasts.
