# Supplementary material for: Rodent maze studies: from following simple rules to complex map learning
Source: Brain Struct Funct. 2024 Mar 15;229(4):823–41. doi: 10.1007/s00429-024-02771-x (PMC11004052; doi:10.1007/s00429-024-02771-x)
Supplement: Supplementary file 1 — Supplementary file1 (PDF 377 KB) [file 429_2024_2771_MOESM1_ESM.pdf]

Rodent maze studies: from following simple rules to complex map learning
Supplemental Materials

Kjell Wijnen, Lisa Genzel*, Jacqueline van der Meij*

S1. Reinforcements used in maze studies

The purpose of using mazes in memory research is often to investigate an animal's ability to navigate to one or more locations via the shortest route possible and while doing so, learning the layout of the maze. An important challenge of maze studies is to confirm that the observed behaviour is a result of the animal's memory and not of, for instance, their mental state. If an experimental group shows diverging performance to reach the goal location of a maze, this could be because the intervention affects their movement or motivation and not so much memory per se. Therefore, it is imperative to correctly, and similarly, motivate all animals in maze experiments. In this section several methods of positive and negative reinforcement will be discussed and compared.

S1.1 Positive reinforcement

S1.1.1 Food motivation

In the first maze study of Small (1901), food was placed inside the maze to motivate the rats in finding their way to the middle of the maze. Since then, food placed at the goal location(s) is the most commonly used motivation enhancer in maze experiments. Although this method seems initially a straight-forward form of positive reinforcement, the methodology might vary greatly among different experiments. If the animal has access to *ad libitum* food in the home environment, they might not be incentivised enough to memorise the maze to search for food. Therefore, a prominent method to make the food reward a stronger incentive is to put animals on dietary restriction: "*They were kept hungry enough so that they would set about the task vigorously*" (Small 1901).

Tolman and Honzik (1930a) investigated how receiving a food reward, or the absence of it, affects the maze solving ability of hungry and non-hungry rats. The rats were divided into four groups: 'Hungry reward', 'Hungry non-reward', 'Less hungry reward' and 'Less hungry non-reward'. Rats in the 'Hungry' groups received their rations of food as a proportion of their weight, so that there was an overall decrease of weight. 'Less hungry' animals received a portion of food so that there was an increase of weight. The reward or non-reward distinction was based on when they received this portion of food. 'Reward' animals received their portion of food at the end of the 14-unit T-maze while 'non-reward' animals received it in their home-cage, three hours after training. As expected, the 'Hungry reward' group performed best by making the least errors. The 'Less hungry non-reward' group, as expected, performed worst of all groups. More interestingly, rats in the 'Less hungry reward', and 'Hungry reward' groups performed similarly with intermediate performance.

Initially, these results indicate that rewarded animals learn the maze better since they make fewer mistakes, thus suggesting a direct relationship between rewards received in the maze and learning performance. Notwithstanding, the cognitive map of the non-rewarded animals is never actually tested as there is no incentive to solve the maze quickly. Therefore, Tolman designed a follow-up study involving a new group of hungry animals. These rats, like the previously non-rewarded group, did not receive any rewards during the initial training period. However, starting from the 11th day of training, these hungry rats began to receive rewards. Surprisingly, Tolman found that these rats, who

had experienced a delay in reinforcement, performed at the same level as the initially rewarded animals during subsequent training sessions (Tolman & Honzik 1930b). This experiment, like a study by Blodgett (1929), demonstrated that animals are able to form cognitive maps of their environment, even in the absence of explicit rewards. This phenomenon of animals learning without it being immediately observable was named 'latent learning'.

Over the years, the preference for dietary restriction has steadily been declining because of regulations concerning animal experiments. Currently in the EU, withdrawal of food for 48 hours is seen as a procedure of moderate severity (Directive 2010/63/EU 2010). However, dietary restriction as a whole is currently a subject of debate among scientists. Dietary restriction with sufficient nutrition has shown to positively affect health and lifespan (Green et al. 2022). The benefits of a restricted diet are not new insights. In a study of Goodrick (1984), the 14-unit T-maze performance of aged dietary restricted (DR) rats was tested. While control rats had received *ad libitum* (AL) food for their entire life, the DR group was fed every other day since weaning age. Rats were tested at the age of 50% mortality rate of that group, which would define them being 'aged'. Interestingly, aged DR rats (30 months) made substantially less errors over the 10-day training period than aged AL rats (22 months), and behaved more like young AL rats (6 months). A few years later, a similar experiment was repeated with mice, including both adult (11-15 months) and aged (31-35 months) individuals. Here, the control mice received ~95 kcal/week (which is less than AL) while the DR mice received ~55 kcal/week. Compared to the aged control, and similar to the rats, dietary restricted aged mice performed better in the 14-unit T-maze (Ingram et al. 1987). For both cases, the increase in performance for DR animals cannot be due to heightened motivation for food. That is because all rats, including control, were starved before the experiment to create similar motivation. For the mice, food motivation in the maze was completely abolished for this reason and fear-motivation via a foot shock was chosen instead. Interestingly, DR only shows an effect in aged animals, suggesting that life-long dietary restriction only starts to show benefits later in life.

S1.1.2 Water motivation

Instead of restricting food intake of an animal, water intake could also be restricted. Then, if an animal solves the maze, it would receive a liquid reward. Just like with hunger motivation, this method of thirst motivation has been used in a multitude of studies (e.g., (Dale 1982; Crouzier et al. 2018; Nagy et al. 2020; Rosenberg et al. 2021; Krausz et al. 2023)). Although water restriction is often an effective method to increase incentive, it is generally seen as a more drastic intervention (Directive 2010/63/EU 2010). Additionally, since yet no benefits have been linked to water restriction (unlike for food restriction), it is seen as a less preferred method of positive reinforcement.

S1.1.3 Hiding or resting location

Since rodents are prey animals, they have an innate preference for dark and enclosed spaces. Some mazes use this preference to their advantage and reward the animal with such spaces at the end of the maze (Harrison et al. 2006). Usually this is an end box as seen with the Barnes maze (Barnes 1979; Tachiki et al. 2023). The Barnes maze especially increases the incentive of the animal to find the escape box by making the rest of the maze a brightly lit, open space. Another maze type that has the potential to heighten a rodent's anxiety until it finds the goal location is the watermaze. Here, instead of an end box, the goal location is a platform where the animal can rest. Since swimming takes a considerable amount of energy, there is an assured incentive to find the goal location as soon as possible.

S1.1.4 Social reward

Besides the beforementioned standard methods of positive reinforcement, other creative rewards have sparsely been given. Rodents are social animals who, in the wild, live in large groups (Ebensperger & Hayes 2008). In laboratory experiments, however, these social groups are often very small or non-existent. Therefore, social interaction with other animals could be used as a strong reward. For juvenile animals, a play-reward is an effective way to incentivise maze solving (Humphreys & Einon 1981; Normansell & Panksepp 1990). For adult (male) rodents on the other hand, a sexual reward in the form of intromission has proven to be a highly effective method of reinforcement (Hernández-González et al. 2014). At the same time, even sniffing the presence of a rat of the opposite sex already acts as a sufficient reward (Ware 1968).

S1.2 Negative reinforcement

S1.2.1 Foot shock

Apart from methods of positive reinforcement, there are also several approaches of negative reinforcement. Unlike receiving a reward for reaching the correct goal location, animals would rather receive a punishment for doing the opposite. The most prevalent punishment in rodent maze studies is the foot shock. For this method, the maze is equipped with an electrically conductive floor to which the researcher can apply a brief (repeated) electrical current if the animal walks through the wrong corridor. Although this method has already been used for a long time, a standard procedure has never been developed. The strength of the foot shock varies greatly between experiments (0.2 mA to 2.5 mA) (Cooper & Krass 1963; Overton 1964; Overton 1968; Farr et al. 1995). Because of this, it is hard to compare or combine results from different experiments. Ethically, the foot shock is not considered a preferable method of reinforcement and in the EU it is even considered a severe intervention (Directive 2010/63/EU 2010).

S1.2.2 Confinement

Another way to punish an animal, if it enters the wrong location, is to confine the animal to that location for a specific amount of time. This way, the animal cannot simply turn around and move towards the correct goal location. For this to be an actual incentive, it should be combined with a reward at the correct goal. Therefore, the animal will experience a greater delay to receiving the reward, if it makes an incorrect decision in the maze. Typically, this method is not used in mazes that contain many dead ends, but rather with mazes that have multiple possible goal locations (Burtt 1916; Ainge et al. 2007).

S1.3 Summary

While traditionally many maze studies just use one reward and/or punishment in the setup, there are now multiple mazes that allow the use of a combination of reinforcements. The Hamlet maze (Crouzier et al. 2018), for instance, uses multiple reinforcements (i.e., food, water, hiding place, running wheel and interaction with strange male/female mouse) at the end of different paths of the maze. Whether to use a positive or negative reinforcement, to use one or more rewards or punishments, or even to use a combination of both, will not only depend on the type of maze selected for the study but also on the research question that is being investigated.

S2. The use of mazes in combination with observational and interventional techniques

The earliest maze studies were aimed at purely examining the navigational behaviour of rodents in a spatial environment. Though some manipulation of the senses, like enucleation, clipping of whiskers and prevention of odour and sound input, were utilized in order to examine their role in

navigation. It was only with the more recent technological developments that maze studies were combined with the use of different observational and interventional techniques.

S2.1 Observational techniques

While the initial maze studies made use of one or more observants to document the rodents' behaviour and performance in the different maze setups, it was Watson (1914) who first described a maze setup in combination with a camera lucida in order to observe the behaviour of rodents in his circular maze from afar. It would not be until 1931, when Dashiell & Bayroff made use of a camera installed above their maze, that maze experiments would be recorded for later analysis. Nowadays, video recording of rodent behaviour in a maze is the standard and can be done both in light as well as in dark conditions (e.g., by use of infrared cameras or LED light on the animal). In addition, the rodents' movements through the maze can be tracked with great detail, both offline as well as online, with the help of a commercial or custom written tracking program.

Besides observing the rodents' behaviour in the maze, the desire for understanding the brain's role in this behaviour caused an increase of the use of electrophysiological techniques (both tethered as well as wireless) in combination with maze studies.

S2.2 Interventional techniques

Whilst electrophysiological recordings present a view on which brain regions might be (in)active when the rodent navigates a maze, they do not provide a conclusive answer on the importance of particular brain regions in both maze learning and memory. In order to examine the detailed role a specific brain region plays in maze navigational behaviour, several interventional techniques can be used. One of the earliest techniques used was lesioning or even removal of a particular brain region. Since this is a permanent intervention, meaning that it can only be done once during the experiment, the need arose for temporary interventions that could be used repeatedly throughout the experiment. Pharmacological interventions, for instance the infusions of a specific drugs, can be utilized to temporarily inhibit a specific region while the rodent encodes or retrieves information in the maze. A more targeted approach, that can both cause temporary inhibition as well as excitation of neurons in a specific brain region, is optogenetics. Both of these techniques thus offer the researcher the option to manipulate certain brain regions during different phases of maze learning.

S2.3 Compatibility with maze studies

The choice of which observational or interventional technique to use not only depends on the specific research question under investigation, but is also influenced by the compatibility of that technique with the maze used in the study. Tethered electrophysiological recordings or optogenetic intervention can for instance only be used in combination with a relatively small, two-dimensional maze. In contrast, the numerous advances in wireless setups opens the possibility to combine these techniques with larger, more naturalistic maze settings as well as with three-dimensional mazes. In addition, the advances made in water-proving make it possible to even combine some of these techniques with a watermaze setup.

S2.4 Summary

In conclusion, besides the nowadays standard use of video recordings of maze studies, one has the possibility to choose from a wide range of other observational as well as interventional techniques to study the neurophysiological underpinnings of rodents' navigational behaviour in any type of maze setup. Which technique would be optimal to use highly depends on the research aim in question as well as the suitable technique/maze setup combination.

Table 1. Maze configurations

Reference	Model	Sex	N	Maze(s) used	Maze size	Maze floor type	Walls	Tested in light/dark	Number of start locations	Number of goal locations
Small (1901)	rat (wild brown & tame albino)	m/f	5 total (3 wild, 2 tame), possible more	Hampton Court maze	1.8x2.4m	wood/wire covered in sawdust	wire netting walls	dark (tested at night)	1	1
Watson (1907)	rat (white)	m/f	?	Hampton Court maze	1.5x2.1m	wood	yes, top covered with wire netting	both	1	1
Carr and Watson (1908)	rat (white)	m/f	8 (3 normal males, 2 blinded males, 3 normal female)	Altered Hampton Court maze	3.7x1.8m	wood	yes	light	1 (training), 3 (test)	1
			7 (3 normal males, 1 blinded male, 3 normal female)	Altered Hampton Court maze	2.9x1.8m	wood	yes	light	?	1
Bogardus and Henke (1911)	rat (white, whiskers removed)	m/f	5	Altered Hampton Court Maze (food box in corner)	?	?	yes, top covered by glass	?	1	1
	rat (white, blinded and pre-trained)	f	6	Altered Hampton Court Maze (food box in corner)	?	?	yes, top covered by glass	?	1	1
	all rats from earlier experiments	m/f	11	Altered Hampton Court Maze (food box in corner)	?	?	yes, top covered by glass	?	1	1
Hicks (1911)	rat (white)	?	17	Hampton Court maze	?	?	?	?	1	1
Watson (1914)	n/a	n/a	n/a	Circular maze	1.77m2 (1.5m in diameter)	wood	yes, aluminium	light	1	1
Vincent (1915)	rat (white)	?	?	Hampton Court maze	?	wood	yes/no	light	1	1
Burtt (1916)	rat (white, inbred & outbred)	m/f	5 total (3 outbred, 2 inbred)	Multiple choice maze	0.9x0.8m	covered in sawdust	yes	light	1	1

Table 1. Maze configurations

Reference	Model	Sex	N	Maze(s) used	Maze size	Maze floor type	Walls	Tested in light/dark	Number of start locations	Number of goal locations
Carr (1917)	rat	?	84 in total divided over multiple mazes	Hampton Court maze	?	?	yes/no	?	1	1
	rat	?	84 (data from Vincent ….)	Circular maze	?	?	?	?	1	1
Hunter (1920)	rat	?	7	Simple alternation, T-maze	?	?	yes	?	1	1
	rat	?	19	Double alternation, T-maze	?	?	yes	?	1	1
	rat	?	6	Double alternation, temporal maze/spatial maze (multiple T-maze)	?	?	yes	?	1	1
	rat	?	7	Simple alternation, temporal maze	?	?	yes	?	1	1
Lashley (1920)	rat	?	60 normal, 127 lesioned	Repeated T-maze (5 different sizes) – simple alternation	?	pinewood	yes, top covered by mesh wire	?	1	1
Tolman (1925)	rat	?	?	T-maze	?	?	?	?	1	1
Helson (1927)	rat (white)	?	4	Compartment maze	1.4x0.3m	?	yes	light	1	1
Miles (1927)	rat	n/a	n/a	Skeleton maze (Narrow-Path Elevated Maze-repeated T-maze)	various sizes	redwood	no	light	1	1
Stone and Nyswander (1927)	rat	m/f	205	Multiple T-maze	1.1x0.8m	linoleum	yes, wood	light	1	1
Elliott (1928)	rat	m	25 (30 control group)	Repeated T-maze (14 units)	1.3x1.7m	?	yes	light	1	1
Stone (1928)	rat (albino)	m/f	201	Modified Carr maze	1.2x1.2m	linoleum	yes	light	1	1

Table 1. Maze configurations

Reference	Model	Sex	N	Maze(s) used	Maze size	Maze floor type	Walls	Tested in light/dark	Number of start locations	Number of goal locations
Yoshioka (1928)	rat (Wistar)	m/f	297	Triangular mazes	various sizes	galvanized iron	yes	light	1	1
Blodgett (1929)	rat (black and white)	m/f	36, 36, 25, 10, 23, 21	Repeated T- and Y-maze, two-way maze	various sizes	wood	yes, top covered with mesh wire	?	1	1
Dennis (1929)	rat (Wistar), blinded and whiskers removed	?	10 (five per group)	Simple rectangular maze with two removable cross walls	0.9x1.3m	wood and brass	yes (cross walls could be lifted)	light	1	1
	rat (Wistar), blinded and whiskers removed	?	8	Simple rectangular maze with one removable cross wall (modified)	0.9x1.3m	wood and brass	yes (cross walls could be lifted)	light	1	1
	rat (Wistar), whiskers removed	?	3	Simple rectangular maze with one removable cross wall (modified)	0.9x1.3m	wood and brass	yes (cross walls could be lifted)	light	1	1
Hunter (1929a)	rat (Wistar), blinded and whiskers removed	?	6	Double alternation bidimensional spatial maze B	?	wood with brass strips	yes	?	1	1
	rat (Wistar), blinded and whiskers removed	?	6 + 3 new	Double alternation bidimensional spatial maze A	?	wood with brass strips	yes	?	1	1
	rat (Wistar), blinded and whiskers removed	?	4 (trained on other mazes)	Simple alternation tridimensional maze (8 units)	?	wood	no	?	1	1
	rat (Wistar)	?	5 new	Simple alternation tridimensional maze (4 units)	?	wood	no	?	1	1
	rat (Wistar), some later blinded	?	24 new	Double alternation tridimensional maze (4 units, rrll)	?	wood	no	light	1	1

Table 1. Maze configurations

Reference	Model	Sex	N	Maze(s) used	Maze size	Maze floor type	Walls	Tested in light/dark	Number of start locations	Number of goal locations
	rat (Wistar), some later blinded	?	11 new	Double alternation tridimensional maze (4 units, llrr)	?	wood	no	light	1	1
	rat (Wistar)	?	5 (trained on previous maze)	Double alteration temporal maze	?	wood	no	?	1	1
Hunter (1929b)	rat (white)	?	34	Tridimensional maze (with simple or double alternation)	0.5x0.5x…m	wood	no	light	1	1
Husband (1929)	rat & human	?	43 rats, 20 humans	Multiple U-maze	9.1x1.8m	wood with roofing paper	yes	light	1	1
Tolman et al. (1929)	rat	n/a	n/a	Self-recording repeated T-maze (17 units)	4.8x2.9m	wood	yes	?	1	1
Trueblood (1929)	rat (white)	n/a	n/a	Tunnel maze	1.3x1.2m	glass	yes	light (from bottom of maze)	1	4
Warden (1929a, 1929b)	n/a	n/a	n/a	Warden-Warner maze (multiple Y-maze)	various sizes	metal/linoleum/ glass	yes	light (from bottom of maze)	1	1
Liggett (1930)	rat (white)	?	25	Sectional maze	approx. 2.4x0.6m	?	yes	light	1	1
McFarlane (1930)	rat (albino)	m/f	approx. 120	Repeated T-maze (dry or filled with water)	1.4x1.5m	tin sheet/water	yes, wood	light	1	1
Miles (1930)	rat (hooded)	m	38 (4 for initial testing)	Skeleton maze	various sizes	redwood	no	light	1	1
Tolman and Honzik (1930a)	rat (white, black, hooded)	m	144	Repeated T-maze (14 units)	2.4x2.4m	hardware cloth	yes	?	1	1
Tolman and Honzik (1930b)	rat (white, black, hooded)	m	82	Repeated T-maze (14 units)	2.4x2.4m	hardware cloth	yes	?	1	1

Table 1. Maze configurations

Reference	Model	Sex	N	Maze(s) used	Maze size	Maze floor type	Walls	Tested in light/dark	Number of start locations	Number of goal locations
Walton (1930)	rat (Wistar)	?	10	Sectional maze	?	galvanized iron	yes	light	1	1
Dashiell and Bayroff (1931)	rat (white)	?	28	Multiple U-maze	?	?	?	?	1	1
	rat (Wistar)	?	11 new	Alternation tridimensional maze (rrllr)	?	wood	no	light	1	1
Gilhousen (1931)	rat	?	7	Elevated open maze	4.6x0.6m	?	partially	?	1	1
Haney (1931)	rat (albino)	f	60 (total)	Repeated T-maze (14 units), rectangular maze as control	1.5x1.9m	metal strips	no, but covered with netting	light	1	1
Krechevsky (1932)	rat	?	40	Multiple-unit discrimination box	?	?	yes	?	1	1
Buel (1934)	rat (albino)	f	27	8-unit Linear maze (multiple T-mazes in a row)	Approx. 4.5x...m	redwood	yes	light	1	1
Maurer and Carr II (1935)	rat	m/f	157	Carr maze (multiple versions)	1.3x1.2m	?	yes	?	1	1
Snygg (1935)	rat (white and black-hooded)	m/f	91	Multiple U-maze	?	?	yes	?	1	2
Biel (1940)	rat (white/albino)	m/f	115 (58 male, 57 female)	Multiple T-watermaze	1.2x1.3m	iron	yes	light	1	1
Hebb and Williams (1946)	rat	?	30	Enclosed field and elevated path maze	0.75x0.9m & 1.2mx1.2m	kitchen table/ plywood	yes/no	light	1	1
Tolman et al. (1946)	rat (Tryon 'bright' & 'dull')	f	56 (30 'bright' and 26 'dull'; all previously trained on Tryon automatic maze)	Sunburst maze	2.3x3.5m	wood	partially	light (at goal location only)	1	1

Table 1. Maze configurations

Reference	Model	Sex	N	Maze(s) used	Maze size	Maze floor type	Walls	Tested in light/dark	Number of start locations	Number of goal locations
Gentry et al. (1947)	rat (Wistar/albino)	?	71 (44 previous training on H-maze)	Sunburst maze	2.3x3.5m	wood	partially	light	1	1
		?	22 (naive)	Sunburst maze	2.3x3.5m	wood	partially	light (at goal location only)	1	1
		?	27 (naive)	Sunburst maze	2.3x3.5m	wood	partially	light (at goal location only)	1	1
		?	18 (naive)	Sunburst maze	2.3x3.5m	wood	partially	light (at goal location only)	1	1
		?	103 (naive)	Sunburst maze	2.3x3.5m	wood	partially	light (at goal location only)	1	1
Gentry et al. (1948)	rat (Wistar/albino/ hooded)	?	80	Multiple T-maze & radial arm maze	3.0x2.1m & 2.3x2.3m	wood	no	light (at goal location only)	1	1
Kendler and Gasser (1948)	rat (Wistar/albino)	m	48	T-maze & radial maze	1.6x1.6m	hardware cloth	yes	light	1	1
Ritchie (1948)	rat (pigmented)	m	50	Sunburst maze	2.7x3.9m	wood	partially	light (at goal location only)	1	1 out of 2
Cooper and Krass (1963)	rat (Lister hooded)	f	32	Enclosed field maze	0.75x0.9m	plywood with aluminium floor	yes	light	1	1
Calhoun (1963)	rat (Brown Norway)	m/f	?	Outside pen	30x30m	wood/metal/ plastic	partially	both	n/a	n/a
Overton (1964)	rat (hooded)	m	151	W-maze	1.07x0.46m	?	yes	?	1	1
Overton (1968)	rat (hooded)	m	37	W-maze	1.07x0.46m	?	yes	?	1	1
		m	22	W-maze	1.07x0.46m	?	yes	?	1	1
		m	34	W-maze	1.07x0.46m		yes		1	1

Table 1. Maze configurations

Reference	Model	Sex	N	Maze(s) used	Maze size	Maze floor type	Walls	Tested in light/dark	Number of start locations	Number of goal locations
Dirlam (1969)	rat (hooded)	m	26	Plus maze	0.3x0.3m	?	?	light	1	n/a
Olton and Samuelson (1976)	rat (albino)	m	6, 6, 12	8 radial arm maze	2.0x2.0m	wood	yes	light	1	8 (all arms need to be visited)
Olton et al. (1977)	rat (albino)	m	17	17 radial arm maze	2.3x2.3m	wood	yes	?	1	17 (all arms need to be visited)
Barnes (1979)	rat (Long-Evans)	?	64	Barnes Maze (circular)	1.22m in diameter	wood	no	light	1	1
Harley (1979)	rat (Sprague-Dawly)	m	47, 36	Modified sunburst maze	approx. 1.05x0.84m	craft paper	yes, plexiglass	?	1	1
Goodale and Dale (1981)	rat (Long-Evans)	m	21	8 radial arm maze	1.0x1.0m	wood	no	dim light	1	8 (all arms need to be visited)
Humphreys and Einon (1981)	rat (Lister hooded)	m/f	12	T-maze	roughly 1x0.5m	perspex tubing	yes (tubes)	dim light	1	1 of 2
Morris (1981)	rat (Lister hooded)	m	33, 16	Watermaze	1.0m in diameter	opaque water	yes	light	multiple	1
Dale (1982)	rat (Long-Evans)	m	22	8 radial arm maze & 8 parallel arm maze	1.9x1.9m & 1.0x0.9m	plywood	partially	dark with dim fluorescent light	1	8 (all arms need to be visited)
Hulse and O'Leary (1982)	rat (Sprague-Dawly)	f	12	4 radial arm maze (plusmaze?)	roughly 3x3m	wood	no (low rim only)	fluorescent light	1	4 (all arms need to be visited in the correct order)
Goodrick (1984)	rat (Wistar)	m	30	Repeated T-maze (14 units)	?	?	yes	?	1	1
Morris (1984)	rat (hooded)	m/f	60	Watermaze	1.3m & 2.1m in diameter	opaque water	yes	light	multiple	1

Table 1. Maze configurations

Reference	Model	Sex	N	Maze(s) used	Maze size	Maze floor type	Walls	Tested in light/dark	Number of start locations	Number of goal locations
Ingram et al. (1987)	mouse (C3B10RF)	f	29	Repeated T-maze (14 units)	?	?	yes	?	1	1
Kollner et al. (1988)	rat (pups)	m/f	48	W-maze	?	?	yes	?	1	1 of 2
Normansell and Panksepp (1990)	rat (Long-Evans)	?	30	T-maze	1.3m length	plywood	yes	dim light	1	?
Grobéty and Schenk (1992)	rat (hooded)	m	18 & 6	3D maze	1.2x1.2x0.1m & 0.8x0.8x0.8m	plastic	yes, tubes	light	4 or 6	1
Farr et al. (1995)	mouse (CD-1)	m/f	30/56	T-maze	0.45x0.46m	plastic	yes		1	1
Levin et al. (1997)	rat	?	?	16 radial arm maze	?	?	yes	?	1	?
Steele and Morris (1999)	rat (Lister hooded)	m	21	Watermaze	2m in diameter	opaque water	yes	light	multiple	multiple (DMP)
Durkin et al. (2000)	mouse (C57BL/6J)	m	16	5-arm maze	roughly 0.65m length	opaque grey PVC	yes	dim light	1	1
Frank et al. (2000)	rat (Long-Evans)	m	4	W-maze, U-maze	0.75m length	?	yes	?	1	multiple (visit arms in correct order)
Poucet and Herrmann (2001)	rat (Long-Evans)	m	17	Elevated open maze	2x3m	plywood	no	light & dark	1	0
Schmitzer-Torbert and Redish (2002)	rat (Brown-Norway-cross)	?	5	Multiple T-maze (5T)	1.4x2.1m	carpet	no	?	1	1

Table 1. Maze configurations

Reference	Model	Sex	N	Maze(s) used	Maze size	Maze floor type	Walls	Tested in light/dark	Number of start locations	Number of goal locations
Harrison et al. (2006)	mouse (B6C3F1/J)	m/f	32	Barnes maze	0.9m in diameter	acrylic	no	light	1	1
Hellweg et al. (2006)	mouse (wild-type and B6-Tg(ThylAPP)23Sdz (APP23))	?	20 (10 each)	"Complex maze" and eight-arm radial	?	gray rigid PVC	?	light (red)	1	1 (radial arm maze food at end of all arms)
Rondi-Reig et al. (2006)	mouse (NR1 knock-out + control)	m	36 (knockout), 52 (control)	Star watermaze	$3.3m^2$ (2.04m in diameter)	opaque water	yes	light	max. 5	max. 5
Ainge et al. (2007)	rat (Lister hooded)	m	5	Repeated Y-maze	1.6x2.0m	wood	yes	light	1	2 out of 4 at the time
	rat (Lister hooded), pre-exposed to different maze	m	10 (6 lesion, 4 control)	Repeated Y-maze	1.6x2.0m	wood	yes	light	1	2 out of 4 at the time
				Right side double Y-maze	1.6x1.0m	wood	yes	light	1	1 out of 2
Roberts et al. (2007)	rat (Long-Evans)	m	30/24	Crossmaze	1.2x1.2m	different textures	yes	light	4	1
Alvernhe et al. (2008)	rat (Long-Evans black hooded)	m	9	M-shaped runway	0.8x0.8m	wood	yes	light	1	2 (other ends of linear alleyway)
Derdikman et al. (2009)	rat (Long-Evans)	m	16	Multiple compartment hairpin maze	1.5x1.5m	linoleum	yes (transparent or opaque)	light	1	2 (each end of the maze)
Dupret et al. (2010)	rat (Long-Evans)	m	7	Cheeseboard maze	1.2m in diameter	?	No	?	1	multiple
Pol-Bodetto et al. (2011)	rat (Long-Evans)	m	219	Double-H watermaze	1.6x1.6m	plexiglass filled with opaque water	yes (transparent)	light	2	1

Table 1. Maze configurations

Reference	Model	Sex	N	Maze(s) used	Maze size	Maze floor type	Walls	Tested in light/dark	Number of start locations	Number of goal locations
Alvernhe et al. (2011)	rat (Long-Evans black hooded)	m	13	Two C-shaped alleyways connected to a linear centre alleyway (adapted from Tolman 1930)	1.3x1.3m	wood	yes	light	1	3 (other ends of M shape)
Yoder et al. (2011)	rat (Long-Evans)	f	8	14-unit T-maze, multi-room maze	?	grey-painted wood	yes	light & dark	1	1
Bett et al. (2012)	rat (Lister hooded)	m	11 (5 shame, 6 lesions)	Double Y-maze	1.0x2.0m	wood	yes	light	1	1
				VD chamber (2 or 3 choices)	1.0x1.0m	wood	yes	light	1	1
Faizi et al. (2012)	mouse (C57BL/6J)	m	133	Barnes maze (40 holes)	1.2m in diameter	?	no	light	1	multiple
Nikbakht et al. (2012)	rat (Wistar)	m	44	8 and 12 radial arm maze	1.8m in diameter	wood/plexiglass	yes	?	1	multiple
Fouquet et al. (2013)	mouse (C57BL/6J)	m	24 (Fos imaging) and 47 (lesion, 25 sham)	Star watermaze	$3.3m^2$ (2.04m in diameter)	opaque water	yes	light	1 training, 1 test	1
Hernández-González et al. (2014)	rat (Wistar)	m	24	T-maze	roughly 1x0.5m	polyurethane-sealed wood	yes	?	1	1
McNamara et al. (2014)	mouse (DAT-IRES-Cre+/-, PV-IRES-CRE+/-)	m	4, 5	Crossword maze	0.95x095m	wood?	no (low rim only)	light	2 out of 4	1
Wilson et al. (2015)	mouse (C57BL/6J)	m	40	Three-dimensional radial maze ("radiolarian" maze), classical radial maze, hexagon maze	0.6x0.6m	crèpe bandage	yes	light	1	multiple
Grieves et al. (2016)	rat (Lister hooded)	m	12 (8 implanted)	Y-T maze	1.4x1.2m	wood	yes	dark	1	3
Olson et al. (2017)	rat (Sprague-Dawly)	m	3	Triple T-maze	1.6x1.25m	?	no (low rim only)	light & dark	1	4

Table 1. Maze configurations

Reference	Model	Sex	N	Maze(s) used	Maze size	Maze floor type	Walls	Tested in light/dark	Number of start locations	Number of goal locations
Tanila et al. (2018)	rat (Long-Evans)	m	4	Townmaze	1.8x1.4m	matte black plastic	yes	?	2	1
Crouzier et al. (2018)	mouse (C57BL/6J)	m/f	320 males and 24 females	Hamlet maze	$2m^2$	black or white infrared-transparent plexiglass	yes	light	1	1
	mouse (Swiss OF-1)	m/f	88 males and 26 females	Hamlet maze	$2m^2$	black or white infrared-transparent plexiglass	yes	light	1	1
Hasz and Redish (2018)	rat (Brown Norway, Harlan)	m	7	Two-stage choice task; double T-maze	?	?	yes	light	1	2
Kapellusch et al. (2018)	Rat (Fischer 344)	m	40	W-maze	1.5x0.9m	acrylic	yes	?	1	1
Rama et al. (2018)	rat (Wistar/ST)	m	2	Multiple Y-maze	1.7x2.1m	transparent PVC sheet	no (low rim only)	light	1	5
Sato et al. (2018)	rat (Long-Evans)	m	8	Lattice maze	1.8x1.8m	black polyvinylchloride	yes	light	3	max. 4
Wood et al. (2018)	rat (Lister hooded)	m	27	Honeycomb maze	1.5m in diameter	ABS plastic	no	light	1 of 37	1 of 37
Illouz et al. (2020)	mouse (C57bl/6)	m	10	Modified Barnes maze	1.2m in diameter	perplex	no	light	1	1 of 40
Mei et al. (2020)	mouse (C57BL/6J)	f	12	8 radial arm maze	1m in diameter	metal/plexiglass	yes, clear tubes	light	1	8
Nagy et al. (2020)	rat (Wistar)	m/f	32 total (16 m, 16f)	Repeated Y-maze	2.7x1.6m	white opaque plexiglass	yes (covered with wired mess)	light (low intensity)	1	1
Alonso et al. (2021)	mouse (C57BL/6J)	m	20	HexMaze	2.0x2.0m	plexiglass	yes	light	24 possible	24 possible

Table 1. Maze configurations

Reference	Model	Sex	N	Maze(s) used	Maze size	Maze floor type	Walls	Tested in light/dark	Number of start locations	Number of goal locations
Mifflin et al. (2021)	mouse (APP/PS1)	m/f	38	Watermaze	1.5m in diameter	opaque water	yes	light	multiple	multiple
Rosenberg et al. (2021)	mouse (C57BL/6J)	m/f	19	Multiple T-labyrinth (64 T-units)	0.6x0.6m	sheet of infrared-transmitting acrylic	yes (covered with clear acrylic ceiling)	light	1	1
de Cothi et al. (2022)	rat (Lister hooded), human and RL agents	m (rats), m/f (humans)	18 humans, 9 rats (6 naive, 3 previously trained on different maze)	Tartarus maze	2.0x2.0m	medium density fibrewood	no	light	multiple	1
Filatova (2022)	rat (Wistar, DAT-HET)	m	10 each	Transformer maze	0.4x0.4m/ 0.6x0.6m	opaque white plexiglass	yes	light	1	4
Tachiki et al. (2023)	mouse (C57BL/6J)	m	111	Barnes maze	1m or 3m in diameter	?	no	light	1	1 out of 12
Krausz et al. (2023)	rat (Long-Evans)	m/f	10	Triangle hex maze	1.3x1.1m	?	yes	light	up to 3	3
Van der Meij et al.	rat	m		HexMaze	9x5m	sawdust	yes	light	96 possible	96 possible

Table 2. Maze methods

Reference	Type of learning	Ego vs allo	Research aim	Negative reinforcement	Positive reinforcement	Deprivation	Combined with technique?	Tracking
Small (1901)	Path learning	ego	navigation, (long-term) memory, previous experience	no	milk & bread	yes	no	observation
Watson (1907)	Path learning	ego	role of kinaesthetic and other senses in maze navigation	no	milk soaked bread and piece of cream cheese	yes	no	observation
Carr and Watson (1908)	Path learning	ego	orientation	no	food	?	no	observation (2-3 observers)
	Path learning	ego	shortened/lengthened paths	no	food	?	no	observation (2-3 observers)
Bogardus and Henke (1911)	Path learning	ego	maze learning and function of tactual sensation in learning	no	food	?	no	observation
	Path learning	ego	maze learning and function of tactual sensation in learning	no	food	?	no	observation
	Path learning	ego	effects of previous maze experiences upon subsequent behavior in slightly altered conditions	no	food	?	no	observation
Hicks (1911)	Path learning	ego	not behaviour, rather types of measuring the behaviour (time, errors, distance)	no	?	yes	no	observation
Watson (1914)	Path learning	ego	n/a	no	food	n/a	n/a	camera lucida
Vincent (1915)	Path learning	ego	role of different senses in solving the maze problem	no	?	?	no	observation
Burtt (1916)	Rule learning	ego	rule learning	yes, confinement in compartment of wrong choice for 5sec	cold boiled potato or green corn	yes	no	observation
Carr (1917)	Path learning	ego	distribution and elimination of error made in maze	no	food	?	no	observation
	Path learning	ego		no	food	?	no	observation
Hunter (1920)	Rule learning	ego	kinaesthesic processes, habit	electric shocks	food	?	no	observation

Table 2. Maze methods

Reference	Type of learning	Ego vs allo	Research aim	Negative reinforcement	Positive reinforcement	Deprivation	Combined with technique?	Tracking
	Rule learning	ego		electric shocks	food	?	no	observation
	Rule learning	ego	double alternation behaviour learned on one maze to be transferred to new maze	no	food	?	no	observation
	Rule learning	ego		no	food	?	no	observation
Lashley (1920)	Rule learning/ Path learning	ego	cerebral function in maze learning	no	food	yes	cerebral lesions	observation
Tolman (1925)	Rule learning	ego	?	?	?	?	?	?
Helson (1927)	Rule learning	ego	insight	yes, electric shock upon entering the wrong compartment	food	yes	no	observation
Miles (1927)	Path learning	ego	n/a	?	food	?	no	observation
Stone and Nyswander (1927)	Path learning	ego	reliability of learning scores in maze	no	food	yes	no	observation
Elliott (1928)	Rule learning/ Path learning	ego	effect of change of reward on maze performance	no	bran maze, sunflower seed	yes	no	observation
Stone (1928)	Path learning	ego	learning, relearning	no	food	yes	no	observation
Yoshioka (1928)	Path learning	ego	discrimination of maze patterns	no	food	?	no	observation
Blodgett (1929)	Rule learning/ Path learning	ego	effect of reward on maze performance	no	food	yes	no	observation
Dennis (1929)	Path learning	ego	role of proprioception in maze habit	yes, electric shock upon touching the cross wall	food	yes	no	camera lucida
	Path learning	ego	role of proprioception in maze habit	yes, electric shock upon touching the cross wall	food	yes	no	camera lucida
	Path learning	ego	role of proprioception in maze habit	yes, electric shock upon touching the cross wall	food	yes	no	camera lucida
Hunter (1929a)	Path learning	ego	sensory control of behaviour in maze	no	milk & bread (outside of maze)	yes	no	observation

Table 2. Maze methods

Reference	Type of learning	Ego vs allo	Research aim	Negative reinforcement	Positive reinforcement	Deprivation	Combined with technique?	Tracking
	Path learning	ego	sensory control of behaviour in maze + influence sound direction	no	milk & bread (outside of maze)	yes	no	observation
	Rule learning	ego	sensory control of behaviour in maze	no	?	?	no	observation
	Rule learning	ego		no	?	?	no	observation
	Rule learning	ego	sensory control of behaviour in maze	no	milk & bread	yes	no	observation
	Rule learning	ego	sensory control of behaviour in maze	no	milk & bread	yes	no	observation
	Rule learning	ego	control for behaviour on double alternation tridimensional maze	alley stop	?	?	no	observation
Hunter (1929b)	Rule learning	ego	combination of characteristics of temporal and spatial maze to test double alteration behaviour	no	food	?	blinding	observation
Husband (1929)	Rule learning/ Path learning	ego	comparisons in learning between animals and human beings	no	food	yes	no	observation
Tolman et al. (1929)	Rule learning	ego	inheritance of maze ability in rats	no	food & water	yes	no	self-recording maze
Trueblood (1929)	Rule learning/ Path learning	ego	n/a	n/a	n/a	n/a	n/a	n/a
Warden (1929a, 1929b)	Rule learning/ Path learning	ego	serial learning	n/a	n/a	n/a	n/a	n/a
Liggett (1930)	Rule learning/ Path learning	ego	elimination of errors	no	food	?	no	observation
McFarlane (1930)	Rule learning/ Path learning	ego	role of kinaesthesis in maze learning	no	food	yes	no	observation
Miles (1927)	Path learning	ego	learning in elevated vs alley maze	no	wet mash	yes	no	observation
Tolman and Honzik (1930a)	Rule learning/ Path learning	ego	role of hunger & reward in maze learning	no	food	yes	no	observation

Table 2. Maze methods

Reference	Type of learning	Ego vs allo	Research aim	Negative reinforcement	Positive reinforcement	Deprivation	Combined with technique?	Tracking
Tolman and Honzik (1930b)	Rule learning/ Path learning	ego	role of reward in maze learning	no	food	yes	no	observation
Walton (1930)	Rule learning/ Path learning	ego	importance of vision in learning and maze running	no	cube of cheese and compressed cottonseed meal	yes	no	observation
Dashiell and Bayroff (1931)	Rule learning	ego	repeated right turn vs alternation	no	food	yes	no	camera
	Rule learning	ego	test influence of final turn on type of errors made	no	milk & bread	yes	no	observation
Gilhousen (1931)	Path learning	ego	insight, short cut	no	food	?	no	observation
Haney (1931)	Rule learning/ Path learning	ego	effect of familiarity on maze performance	no	food	yes	no	observation
Krechevsky (1932)	Rule learning	ego		?	?	?	no	observation
Buel (1934)	Rule learning/ Path learning	ego	spatial navigation	no	Steenbock mash	?	no	observation
Maurer and Carr II (1935)	Path learning	ego	spatial navigation	no	food	?	no	observation
Snygg (1935)	Rule learning/ Path learning	ego	short vs long path to food box	no	food	yes	no	observation
Biel (1940)	Path learning	ego	early age differences in maze performance	no	n/a	n/a	no	observation
Hebb and Williams (1946)	Path learning	ego	animal intelligence / spatial learning	no	food	?	cortical operated?	observation
Tolman et al. (1946)	Path learning	ego/ allo	orientation, short cut, path in direction of goal	no	wet food	yes	no	observation
Gentry et al. (1947)	Path learning	ego/ allo	role of light cues in navigation/learning	no	food (dog chow?)	yes	no	observation

Table 2. Maze methods

Reference	Type of learning	Ego vs allo	Research aim	Negative reinforcement	Positive reinforcement	Deprivation	Combined with technique?	Tracking
	Path learning	ego/ allo	lengthened training period	no	food (dog chow?)	yes	no	observation
	Path learning	ego/ allo	role of light cues in navigation/learning	no	food (dog chow?)	yes	no	observation
	Path learning	ego/ allo	role of pretraining maze on navigation behaviour at test	no	food (dog chow?)	yes	no	observation
			natural searching behaviour without training	no	food (dog chow?)	yes	no	observation
Gentry et al. (1948)	Path learning	ego/ allo	role of spatial learning on orientation and ability short cut	no	food (dog chow?)	yes	no	observation
Kendler and Gasser (1948)	Path learning/ direction	ego/ allo	trained on one maze, tested on the other	no	food (mashed dog chow)	yes	no	observation
Ritchie (1948)	Path learning	ego/ allo	role of light cues in navigation/learning	yes, electric shock before change in goal location	food	no	no	observation
Cooper and Krass (1963)	Path learning	ego	effects of drug on spatial memory	yes, electric shock when animal did not perform fast enough	wet mash	yes	drug injection	observation
Calhoun (1963)	Complex map	allo	role of social interaction on population growth	no	food	no	no	observation/ camera
Overton (1964)	Rule learning	ego	ability to distinguish between drug states	yes, electric shock until escaping to the goal box	no	no	drug injection	observation
Overton (1968)	Rule learning	ego	ability to distinguish between drug states	yes, electric shock until escaping to the goal box	no	no	drug injection	observation
	Rule learning	ego	ability to distinguish between drug states	yes, electric shock until escaping to the goal box	no	no	drug injection	observation
	Rule learning	ego	ability to distinguish between drug states	yes, electric shock until escaping to the goal box	no	no	drug injection	observation
Dirlam (1969)	n/a	n/a	effects of brain lesions on social behaviour	no	no	no	lesions	observation
Olton and Samuelson (1976)	Rule learning/ Path learning	ego/ allo	spatial memory	no	food pellets	yes	no	observation

Table 2. Maze methods

Reference	Type of learning	Ego vs allo	Research aim	Negative reinforcement	Positive reinforcement	Deprivation	Combined with technique?	Tracking
Olton et al. (1977)	Rule learning/ Path learning	ego/ allo	spatial memory	no	food pellets	yes	no	observation
Barnes (1979)	Simple map	ego	memory deficits with senescence	no	food	no	tethered ephys recordings	observation
Harley (1979)	Path learning	ego/ allo	role of hippocampus in spatial memory	no	saccharin solution	yes	bilateral hippocampus or neocortex removed	observation
Goodale and Dale (1981)	Rule learning/ Path learning	ego	role of posterior cortex in spatial learning	no	food pellets	yes	bilateral eye enucleations and/or lesions of posterior neocortex	?
Humphreys and Einon (1981)	Rule learning	ego	play as reinforcer on maze learning	no	companionship	no	no	observation
Morris (1981)	Complex map	allo	spatial learning	no	no	no	no	camera
Dale (1982)	Rule learning/ Path learning	ego	role of visual cues on maze performance in sighted and blinded rats	no	water	yes, water	enucleation	observation
Hulse and O'Leary (1982)	Rule learning	ego	categorization, spatial learning	no	crushed food pellet	yes	no	observation
Goodrick (1984)	Path learning	ego	effects of dietary restriction on spatial memory	no	food	yes	no	observation
Morris (1984)	Complex map	allo	spatial learning	no	no	no	lesions	camera
Ingram et al. (1987)	Path learning	ego	effects of dietary restriction on spatial memory	yes, electric shock upon entering the wrong compartment	no	no	no	observation
Kollner et al. (1988)	Rule learning	ego	spatial (reversal) learning	yes, electric shock upon entering the wrong compartment	?	?	no	observation
Normansell and Panksepp (1990)	Path learning	ego	opiates and play reward	no	companionship	no	drug injections	observation
Grobéty and Schenk (1992)	Simple map	allo?	spatial learning in 3D	no	condensed milk	yes	no	camera

Table 2. Maze methods

Reference	Type of learning	Ego vs allo	Research aim	Negative reinforcement	Positive reinforcement	Deprivation	Combined with technique?	Tracking
Farr et al. (1995)	Path learning	ego	spatial learning on hormone treatment	yes, electric shock upon entering the wrong compartment	no	n/a	hormone treatment	observation
Levin et al. (1997)	Rule learning	allo	effect of nicotine on memory	?	?	?	drug injections	?
Steele and Morris (1999)	Complex map	allo	role of NMDA on DMP	no	no	no	infusions/lesions	camera
Durkin et al. (2000)	Rule learning/ Path learning	ego	visuo-spatial attention and spatial working memory	no	food pellet	yes	no	observation
Frank et al. (2000)	Rule learning	ego	spatial navigation	no	liquid chocolate	yes	tethered ephys recordings	
Poucet and Herrmann (2001)	n/a	ego	maze exploration in light and dark conditions	no	no	no	no	camera
Schmitzer-Torbert and Redish (2002)	Rule learning/ Path learning	ego	sequence learning	no	food pellet	yes	no	camera + LED
Harrison et al. (2006)	Path learning	allo	spatial and nonspatial escape strategies	no	hiding place	no	no	camera
Hellweg et al. (2006)	Path learning	ego	spatial navigation	no	food pellet	yes	neurotrophin signalling	camera
Rondi-Reig et al. (2006)	Path learning	ego/ allo	navigation strategy	no	no	no		camera
Ainge et al. (2007)	Rule learning	ego	spatial navigation	yes, 5s confinement in non-baited goal box	weetos	yes	tethered ephys recordings	b/w camera + LED
	Rule learning	ego	role of hippocampus in spatial navigation	yes, 5s confinement in non-baited goal box	weetos	yes	hippocampal lesion	b/w camera
	Rule learning	ego	win-stay and lose-shift	yes, 5s confinement in non-baited goal box	weetos	yes	hippocampal lesion	b/w camera
Roberts et al. (2007)	Rule learning/ Path learning	ego/ allo	spatial navigation, short cuts	no	fresh cheese	yes	no	?
Alvernhe et al. (2008)	Rule learning	ego	short cuts	no	food pellet	yes	tethered ephys recordings	camera

Table 2. Maze methods

Reference	Type of learning	Ego vs allo	Research aim	Negative reinforcement	Positive reinforcement	Deprivation	Combined with technique?	Tracking
Derdikman et al. (2009)	Rule learning	ego	short cuts, 'virtual task'	no	crumbs of chocolate cereal	yes	tethered ephys recordings	camera
Dupret et al. (2010)	Path learning/ Complex map	allo?/ ego	hippocampal map reactivation	no	food	no	tethered ephys recordings	camera
Pol-Bodetto et al. (2011)	Path learning	ego/ allo	spatial memory	no	no	no	drug injection	observation
Alvernhe et al. (2011)	Rule learning	ego	short/long detours	no	food pellet	yes	tethered ephys recordings	camera
Yoder et al. (2011)	Path learning	ego	head direction cells	no	sucrose pellet	no	tethered ephys recordings	camera + LED
Bett et al. (2012)	Rule learning	ego	VTE behaviour	yes, 10s confinement in non-baited goal box	weetos	yes	hippocampal lesion	camera
	Rule learning	allo?	non-spatial (non-hpc dep) task to test VTE behaviour	no	weetos	yes	hippocampal lesion	camera + LED
Faizi et al. (2012)	Rule learning	allo	spatial learning in Alzheimer's model	no	escape hole	no	no	observation
Nikbakht et al. (2012)	Rule learning	allo	experience-dependent expression of hippocampal *Arc* and *Homer 1a* after spatial learning	no	popcorn	yes	no	camera
Fouquet et al. (2013)	Path learning	ego/ allo	navigation strategy	no	no	no	hippocampal/ striatum lesions, Fos imaging	camera
Hernández-González et al. (2014)	Path learning	Ego	sexual reward	no	intromission	no	drug injections	observation
McNamara et al. (2014)	Simple map	ego/ allo	role of dopaminergic neurons and hippocampal reactivations in spatial memory	no	condensed milk	?	tethered ephys recordings/ photo-stimulation	camera + LED
Wilson et al. (2015)	Simple map	ego/ allo	spatial learning in 3d	no	condensed milk	yes	no	?
Grieves et al. (2016)	Rule learning/ Path learning	ego/ allo	win-stay, lose-shift	no	cocopops	yes	tethered ephys recordings	camera + LED

Table 2. Maze methods

Reference	Type of learning	Ego vs allo	Research aim	Negative reinforcement	Positive reinforcement	Deprivation	Combined with technique?	Tracking
Olson et al. (2017)	Rule learning/ Path learning	ego/ allo	assess the prevalence of subiculum axis-tuned neurons	no	Cheerios cereal	yes	tethered ephys recordings	camera + LED
Tanila et al. (2018)	Rule learning/ Path learning	ego/ allo	spatial map	less reward	minipellets	yes	tethered ephys recordings	camera + LED
Crouzier et al. (2018)	Simple map	allo? Topographic memory	route learning strategies, impact training on brain plasticity/hippocampal neurogenesis, memory-impairing effects of cholinergic receptor antagonist, spatial disorientation through drugs	no	water/food/interaction/hide/run	yes, water and food + non-deprived controls	FosB/ΔFosB immunolabeling	camera + videotrack
	Simple map	allo? Topographic memory	route learning strategies, impact training on brain plasticity/hippocampal neurogenesis, memory-impairing effects of cholinergic receptor antagonist, spatial disorientation through drugs	no	water/food/interaction/hide/run	yes, water and food + non-deprived controls	FosB/ΔFosB immunolabeling	camera + videotrack
Hasz and Redish (2018)	Rule learning	ego/ allo	decision making	no	food pellet	yes	no	camera
Kapellusch et al. (2018)	Rule learning	ego	learning deficits in spatial alternation	no	diluted vanilla nutrition shake	no	no	IR sensors
Rama et al. (2018)	Path learning	ego	decision making	no	cereal pellets	yes	tethered ephys recordings	camera
Sato et al. (2018)	Simple map	ego/ allo	cognitive map, flexible spatial behaviour, shortcuts, detours	no	food pellet	yes	no	camera
Wood et al. (2018)	Rule learning / Simple map	ego/ allo	hippocampal-dependent spatial navigation	no	cheerio	yes	lesions	observation
Illouz et al. (2020)	Simple map	ego/ allo	spatial learning	no	Hiding place	no	no	observation
Mei et al. (2020)	Rule learning	allo	asses spatial working and combined working/reference memory paradigms	no	sugar pellet	no	no	ID sorter
Nagy et al. (2020)	Simple map	ego/ allo	decision making + collective searching behaviour	no	water	yes, water	no	low light sensitive camera
Alonso et al. (2021)	Complex map	allo	build up and updating of spatial map	no	cocopops	yes	no	camera

Table 2. Maze methods

Reference	Type of learning	Ego vs allo	Research aim	Negative reinforcement	Positive reinforcement	Deprivation	Combined with technique?	Tracking
Mifflin et al. (2021)	Complex map	allo	sex differences in Alzheimer's model	no	no	no	no	camera
Rosenberg et al. (2021)	Simple map	ego/ allo	exploration, decisions, insight	no	water	yes, water	no	camera (underneath the maze)
de Cothi et al. (2022)	Simple map	allo	navigation, predictive maps	no	chocolate milk (human: financial reward)	yes (rats)	no	camera
Filatova (2022)	Simple map	allo	learning, memory and navigation	no	peeled sunflower seed	yes	no	camera
Tachiki et al. (2023)	Simple map	allo	spatial learning	no	escape box	no	some animals received scopolamine hydrobromide injections	camera
Krausz et al. (2023)	Simple map	ego?	cost-benefit decisions in navigation	no	sucrose solution droplets	yes, water	photometry recordings	camera
Van der Meij et al.	Complex map	allo	build up and updating of spatial map	no	weetos	no	yes	12 cameras

References

Ainge, J. A., Tamosiunaite, M., Woergoetter, F., & Dudchenko, P. A. (2007). Hippocampal CA1 place cells encode intended destination on a maze with multiple choice points. *J Neurosci*, *27*(36), 9769-9779. https://doi.org/10.1523/JNEUROSCI.2011-07.2007

Alonso, A., Bokeria, L., van der Meij, J., Samanta, A., Eichler, R., Lotfi, A., Spooner, P., Navarro Lobato, I., & Genzel, L. (2021). The HexMaze: A Previous Knowledge Task on Map Learning for Mice. *eNeuro*, *8*(4). https://doi.org/10.1523/ENEURO.0554-20.2021

Alvernhe, A., Save, E., & Poucet, B. (2011). Local remapping of place cell firing in the Tolman detour task. *Eur J Neurosci*, *33*(9), 1696-1705. https://doi.org/10.1111/j.1460-9568.2011.07653.x

Alvernhe, A., Van Cauter, T., Save, E., & Poucet, B. (2008). Different CA1 and CA3 representations of novel routes in a shortcut situation. *J Neurosci*, *28*(29), 7324-7333. https://doi.org/10.1523/JNEUROSCI.1909-08.2008

Barnes, C. A. (1979). Memory deficits associated with senescence: a neurophysiological and behavioral study in the rat. *J Comp Physiol Psychol*, *93*(1), 74-104. https://doi.org/10.1037/h0077579

Bett, D., Allison, E., Murdoch, L. H., Kaefer, K., Wood, E. R., & Dudchenko, P. A. (2012). The neural substrates of deliberative decision making: contrasting effects of hippocampus lesions on performance and vicarious trial-and-error behavior in a spatial memory task and a visual discrimination task. *Front Behav Neurosci*, *6*, 70. https://doi.org/10.3389/fnbeh.2012.00070

Biel, W. C. (1940). Early Age Differences in Maze Performance in the Albino Rat. *The Pedagogical Seminary and Journal of Genetic Psychology*, *56*(2), 439-453. https://doi.org/10.1080/08856559.1940.10534511

Blodgett, H. C. (1929). The effect of the introduction of reward upon the maze performance of rats. *University of California Publications in Psychology*.

Bogardus, E. S., & Henke, F. G. (1911). Experiments on tactual sensations in the white rat. *Journal of Animal Behavior*, *1*(2), 125-137. https://doi.org/10.1037/h0072495

Buel, J. (1934). The linear maze. I. "Choice-point expectancy," "correctness," and the goal gradient. *Journal of Comparative Psychology*, *17*(2), 185-199. https://doi.org/10.1037/h0072346

Burtt, H. E. (1916). A study of the behavior of the white rat by the multiple choice method. *Journal of Animal Behavior*, *6*(3), 222-246. https://doi.org/10.1037/h0074872

Calhoun, J. B. (1963). *The ecology and sociology of the Norway rat*. US Department of Health, Education, and Welfare, Public Health Service.

Carr, H. (1917). Maze learning with the white rat. II. Blind animals. *Journal of Animal Behavior*.

Carr, H., & Watson, J. B. (1908). Orientation in the white rat. *Journal of Comparative Neurology and Psychology*, *18*(1), 27-44. https://doi.org/10.1002/cne.920180103

Cooper, R. M., & Krass, M. (1963). Strychnine: Duration of the Effects on Maze-Learning. *Psychopharmacologia*, *4*, 472-475. https://doi.org/10.1007/BF00403352

Crouzier, L., Gilabert, D., Rossel, M., Trousse, F., & Maurice, T. (2018). Topographical memory analyzed in mice using the Hamlet test, a novel complex maze. *Neurobiol Learn Mem*, *149*, 118-134. https://doi.org/10.1016/j.nlm.2018.02.014

Dale, R. H. (1982). Parallel-arm maze performance of sighted and blind rats: Spatial memory and maze structure. *Behaviour Analysis Letters*, *2*(3), 127.
Dashiell, J. F., & Bayroff, A. G. (1931). A forwardgoing tendency in maze running. *Journal of Comparative Psychology*, *12*(1), 77-94. https://doi.org/10.1037/h0070769
de Cothi, W., Nyberg, N., Griesbauer, E. M., Ghaname, C., Zisch, F., Lefort, J. M., Fletcher, L., Newton, C., Renaudineau, S., Bendor, D., Grieves, R., Duvelle, E., Barry, C., & Spiers, H. J. (2022). Predictive maps in rats and humans for spatial navigation. *Curr Biol*, *32*(17), 3676-3689 e3675. https://doi.org/10.1016/j.cub.2022.06.090
Dennis, W. (1929). The Sensory Control of the White Rat in the Maze Habit. *The Pedagogical Seminary and Journal of Genetic Psychology*, *36*(1), 59-90. https://doi.org/10.1080/08856559.1929.10532186
Derdikman, D., Whitlock, J. R., Tsao, A., Fyhn, M., Hafting, T., Moser, M. B., & Moser, E. I. (2009). Fragmentation of grid cell maps in a multicompartment environment. *Nat Neurosci*, *12*(10), 1325-1332. https://doi.org/10.1038/nn.2396
Directive 2010/63/EU of the European Parliament and of the Council of 22 September 2010 on the protection of animals used for scientific purposes. (2010).
Dirlam, D. K. (1969). The effects of septal, thalamic, and tegmental lesions on general activity in the hooded rat. *Can J Psychol*, *23*(5), 303-314. https://doi.org/10.1037/h0082818
Dupret, D., O'Neill, J., Pleydell-Bouverie, B., & Csicsvari, J. (2010). The reorganization and reactivation of hippocampal maps predict spatial memory performance. *Nat Neurosci*, *13*(8), 995-1002. https://doi.org/10.1038/nn.2599
Durkin, T. P., Beaufort, C., Leblond, L., & Maviel, T. (2000). A 5-arm maze enables parallel measures of sustained visuo-spatial attention and spatial working memory in mice. *Behav Brain Res*, *116*(1), 39-53. https://doi.org/10.1016/s0166-4328(00)00248-5
Ebensperger, L. A., & Hayes, L. D. (2008). On the dynamics of rodent social groups. *Behav Processes*, *79*(2), 85-92. https://doi.org/10.1016/j.beproc.2008.05.006
Elliott, M. H. (1928). *The effect of change of reward on the maze performance of rats*. University of California Press.
Faizi, M., Bader, P. L., Saw, N., Nguyen, T. V., Beraki, S., Wyss-Coray, T., Longo, F. M., & Shamloo, M. (2012). Thy1-hAPP(Lond/Swe+) mouse model of Alzheimer's disease displays broad behavioral deficits in sensorimotor, cognitive and social function. *Brain Behav*, *2*(2), 142-154. https://doi.org/10.1002/brb3.41
Farr, S. A., Flood, J. F., Scherrer, J. F., Kaiser, F. E., Taylor, G. T., & Morley, J. E. (1995). Effect of ovarian steroids on footshock avoidance learning and retention in female mice. *Physiol Behav*, *58*(4), 715-723. https://doi.org/10.1016/0031-9384(95)00124-2
Filatova, E. (2022). Transformer maze for the evaluation of the learning and memory in rodents. *Heliyon*, *8*(10).
Fouquet, C., Babayan, B. M., Watilliaux, A., Bontempi, B., Tobin, C., & Rondi-Reig, L. (2013). Complementary Roles of the Hippocampus and the Dorsomedial Striatum during Spatial and Sequence-Based Navigation Behavior. *PloS one*, *8*(6), e67232. https://doi.org/10.1371/journal.pone.0067232
Frank, L. M., Brown, E. N., & Wilson, M. (2000). Trajectory encoding in the hippocampus and entorhinal cortex. *Neuron*, *27*(1), 169-178. https://doi.org/10.1016/s0896-6273(00)00018-0
Gentry, G., Brown, W. L., & Kaplan, S. J. (1947). An experimental analysis of the spatial location hypothesis in learning. *J Comp Physiol Psychol*, *40*(5), 309-322. https://doi.org/10.1037/h0061537

Gentry, G., Brown, W. L., & Lee, H. (1948). Spatial Location in the Learning of a Multiple-T Maze. *J Comp Physiol Psychol*, *41*(5), 312-318. https://doi.org/10.1037/h0056159
Gilhousen, H. C. (1931). An Investigation of "Insight" in Rats. *Science*, *73*(1904), 711-712. https://doi.org/10.1126/science.73.1904.711
Goodale, M. A., & Dale, R. H. (1981). Radial-maze performance in the rat following lesions of posterior neocortex. *Behav Brain Res*, *3*(2), 273-288. https://doi.org/10.1016/0166-4328(81)90051-6
Goodrick, C. L. (1984). Effects of lifelong restricted feeding on complex maze performance in rats. *Age*, *7*(1), 1-2. https://doi.org/10.1007/bf02431887
Green, C. L., Lamming, D. W., & Fontana, L. (2022). Molecular mechanisms of dietary restriction promoting health and longevity. *Nat Rev Mol Cell Biol*, *23*(1), 56-73. https://doi.org/10.1038/s41580-021-00411-4
Grieves, R. M., Wood, E. R., & Dudchenko, P. A. (2016). Place cells on a maze encode routes rather than destinations. *Elife*, *5*. https://doi.org/10.7554/eLife.15986
Grobéty, M.-C., & Schenk, F. (1992). Spatial learning in a three-dimensional maze. *Animal Behaviour*, *43*(6), 1011-1020.
Haney, G. W. (1931). The effect of familiarity on maze performance of albino rats. *(No Title)*.
Harley, C. W. (1979). Arm choices in a sunburst maze: effects of hippocampectomy in the rat. *Physiol Behav*, *23*(2), 283-290. https://doi.org/10.1016/0031-9384(79)90369-x
Harrison, F. E., Reiserer, R. S., Tomarken, A. J., & McDonald, M. P. (2006). Spatial and nonspatial escape strategies in the Barnes maze. *Learn Mem*, *13*(6), 809-819. https://doi.org/10.1101/lm.334306
Hasz, B. M., & Redish, A. D. (2018). Deliberation and Procedural Automation on a Two-Step Task for Rats. *Front Integr Neurosci*, *12*, 30. https://doi.org/10.3389/fnint.2018.00030
Hebb, D. O., & Williams, K. (1946). A method of rating animal intelligence. *The Journal of general psychology*, *34*(1), 59-65.
Hellweg, R., Lohmann, P., Huber, R., Kühl, A., & Riepe, M. W. (2006). Spatial navigation in complex and radial mazes in APP23 animals and neurotrophin signaling as a biological marker of early impairment. *Learning & Memory*, *13*(1), 63-71.
Helson, H. (1927). Insight in the white rat. *Journal of Experimental Psychology*, *10*(5), 378-396. https://doi.org/10.1037/h0070577
Hernández-González, M., Aguirre, F. A. R., Guevara, M. Á., Quirarte, G. L., & Magallanes, P. H. (2014). Basolateral Amygdala Inactivation Reduces Sexual Motivation in Male Rats during Performance of a T-Maze Task with a Sexual Reward. *Journal of Behavioral and Brain Science*, *04*(05), 223-233. https://doi.org/10.4236/jbbs.2014.45024
Hicks, V. C. (1911). The relative values of the different curves of learning. *Journal of Animal Behavior*, *1*(2), 138-156. https://doi.org/10.1037/h0074676
Hulse, S. H., & O'Leary, D. K. (1982). Serial pattern learning: Teaching an alphabet to rats. *Journal of experimental psychology: Animal behavior processes*, *8*(3), 260.
Humphreys, A. P., & Einon, D. F. (1981). Play as a reinforcer for maze-learning in juvenile rats. *Animal Behaviour*, *29*(1), 259-270. https://doi.org/10.1016/s0003-3472(81)80173-x
Hunter, W. S. (1920). The temporal maze and kinaesthetic sensory processes in the white rat. *Psychobiology*, *2*(1), 1-17. https://doi.org/10.1037/h0073855
Hunter, W. S. (1929a). The Sensory Control of the Maze Habit in the White Rat. *The Pedagogical Seminary and Journal of Genetic Psychology*, *36*(4), 505-537. https://doi.org/10.1080/08856559.1929.10532211

Hunter, W. S. (1929b). A Tridimensional Maze. *Journal of General Psychology*, *2*, 130-134.
Husband, R. W. (1929). A comparison of human adults and white rats in maze learning. *Journal of Comparative Psychology*, *9*(6), 361-377. https://doi.org/10.1037/h0074679
Illouz, T., Madar, R., & Okun, E. (2020). A modified Barnes maze for an accurate assessment of spatial learning in mice. *J Neurosci Methods*, *334*, 108579. https://doi.org/10.1016/j.jneumeth.2020.108579
Ingram, D. K., Weindruch, R., Spangler, E. L., Freeman, J. R., & Walford, R. L. (1987). Dietary restriction benefits learning and motor performance of aged mice. *J Gerontol*, *42*(1), 78-81. https://doi.org/10.1093/geronj/42.1.78
Kapellusch, A. J., Lester, A. W., Schwartz, B. A., Smith, A. C., & Barnes, C. A. (2018). Analysis of learning deficits in aged rats on the W-track continuous spatial alternation task. *Behav Neurosci*, *132*(6), 512-519. https://doi.org/10.1037/bne0000269
Kendler, H. H., & Gasser, W. P. (1948). Variables in spatial learning; number of reinforcements during training. *J Comp Physiol Psychol*, *41*(3), 178-187. https://doi.org/10.1037/h0057407
Kollner, O., Kollner, U., Gob, R., & Klingberg, F. (1988). Ontogenetic development of avoidance learning in rats after eye opening. *Biomed Biochim Acta*, *47*(12), 985-996. https://www.ncbi.nlm.nih.gov/pubmed/3254159
Krausz, T. A., Comrie, A. E., Frank, L. M., Daw, N. D., & Berke, J. D. (2023). Dual credit assignment processes underlie dopamine signals in a complex spatial environment. *bioRxiv*, 2023.2002. 2015.528738. https://doi.org/10.1101/2023.02.15.528738
Krechevsky, I. (1932). " Hypotheses" in rats. *Psychological Review*, *39*(6), 516.
Lashley, K. S. (1920). Studies of cerebral function in learning. *Psychobiology*, *2*(1), 55.
Levin, E. D., Kaplan, S., & Boardman, A. (1997). Acute nicotine interactions with nicotinic and muscarinic antagonists: working and reference memory effects in the 16-arm radial maze. *Behav Pharmacol*, *8*(2-3), 236-242. https://www.ncbi.nlm.nih.gov/pubmed/9833018
Liggett, J. R. (1930). Apparatus: The Unit Maze: A Study in Maze Method. *The Pedagogical Seminary and Journal of Genetic Psychology*, *37*(1), 163-175.
Maurer, S., & Carr II, H. A. (1935). The empirical determination of maze reliability. *Journal of Comparative Psychology*, *20*(2), 291.
McFarlane, D. (1930). The role of kinesthesis in maze learning. Univ. of Cal. *Publ. Psychol*, *4*.
McNamara, C. G., Tejero-Cantero, A., Trouche, S., Campo-Urriza, N., & Dupret, D. (2014). Dopaminergic neurons promote hippocampal reactivation and spatial memory persistence. *Nat Neurosci*, *17*(12), 1658-1660. https://doi.org/10.1038/nn.3843
Mei, J., Kohler, J., Winter, Y., Spies, C., Endres, M., Banneke, S., & Emmrich, J. V. (2020). Automated radial 8-arm maze: A voluntary and stress-free behavior test to assess spatial learning and memory in mice. *Behav Brain Res*, *381*, 112352. https://doi.org/10.1016/j.bbr.2019.112352
Mifflin, M. A., Winslow, W., Surendra, L., Tallino, S., Vural, A., & Velazquez, R. (2021). Sex differences in the IntelliCage and the Morris water maze in the APP/PS1 mouse model of amyloidosis. *Neurobiol Aging*, *101*, 130-140. https://doi.org/10.1016/j.neurobiolaging.2021.01.018
Miles, W. R. (1927). The Narrow-Path Elevated Maze for Studying Rats. *Experimental Biology and Medicine*, *24*(5), 454-456. https://doi.org/10.3181/00379727-24-3414
Morris, R. (1984). Developments of a water-maze procedure for studying spatial learning in the rat. *J Neurosci Methods*, *11*(1), 47-60. https://doi.org/10.1016/0165-0270(84)90007-4

Morris, R. G. M. (1981). Spatial localization does not require the presence of local cues. *Learning and Motivation*, *12*(2), 239-260. https://doi.org/10.1016/0023-9690(81)90020-5

Nagy, M., Horicsanyi, A., Kubinyi, E., Couzin, I. D., Vasarhelyi, G., Flack, A., & Vicsek, T. (2020). Synergistic Benefits of Group Search in Rats. *Curr Biol*, *30*(23), 4733-4738 e4734. https://doi.org/10.1016/j.cub.2020.08.079

Nikbakht, N., Zarei, B., Shirani, E., Moshtaghian, J., Esmaeili, A., & Habibian, S. (2012). Experience-dependent expression of rat hippocampal Arc and Homer 1a after spatial learning on 8-arm and 12-arm radial mazes. *Neuroscience*, *218*, 49-55. https://doi.org/10.1016/j.neuroscience.2012.05.025

Normansell, L., & Panksepp, J. (1990). Effects of morphine and naloxone on play-rewarded spatial discrimination in juvenile rats. *Dev Psychobiol*, *23*(1), 75-83. https://doi.org/10.1002/dev.420230108

Olson, J. M., Tongprasearth, K., & Nitz, D. A. (2017). Subiculum neurons map the current axis of travel. *Nat Neurosci*, *20*(2), 170-172. https://doi.org/10.1038/nn.4464

Olton, D. S., Collison, C., & Werz, M. A. (1977). Spatial memory and radial arm maze performance of rats. *Learning and Motivation*, *8*(3), 289-314.

Olton, D. S., & Samuelson, R. J. (1976). Remembrance of places passed: spatial memory in rats. *Journal of experimental psychology: Animal behavior processes*, *2*(2), 97.

Overton, D. A. (1964). Statedependent or" dissociated" learning produced with pentobarbital. *Journal of comparative and physiological psychology*, *57*(1), 3.

Overton, D. A. (1968). Visual cues and shock sensitivity in the control of T-maze choice by drug conditions. *J Comp Physiol Psychol*, *66*(1), 216-219. https://doi.org/10.1037/h0025989

Pol-Bodetto, S., Jeltsch-David, H., Lecourtier, L., Rusnac, N., Mam-Lam-Fook, C., Cosquer, B., Geiger, K., & Cassel, J. C. (2011). The double-H maze test, a novel, simple, water-escape memory task: acquisition, recall of recent and remote memory, and effects of systemic muscarinic or NMDA receptor blockade during training. *Behav Brain Res*, *218*(1), 138-151. https://doi.org/10.1016/j.bbr.2010.11.043

Poucet, B., & Herrmann, T. (2001). Exploratory patterns of rats on a complex maze provide evidence for topological coding. *Behavioural Processes*, *53*(3), 155-162.

Rama, E., Capi, G., Fujimura, Y., Tanaka, N., Kawahara, S., & Jindai, M. (2018). Novel Biological Based Method for Robot Navigation and Localization. *Journal of Electronic Science and Technology*, *16*(1), 16-23.

Ritchie, B. F. (1948). Studies in spatial learning. VI. Place orientation and direction orientation. *Journal of Experimental Psychology*, *38*(6), 659.

Roberts, W. A., Cruz, C., & Tremblay, J. (2007). Rats take correct novel routes and shortcuts in an enclosed maze. *J Exp Psychol Anim Behav Process*, *33*(2), 79-91. https://doi.org/10.1037/0097-7403.33.2.79

Rondi-Reig, L., Petit, G. H., Tobin, C., Tonegawa, S., Mariani, J., & Berthoz, A. (2006). Impaired sequential egocentric and allocentric memories in forebrain-specific-NMDA receptor knock-out mice during a new task dissociating strategies of navigation. *J Neurosci*, *26*(15), 4071-4081. https://doi.org/10.1523/JNEUROSCI.3408-05.2006

Rosenberg, M., Zhang, T., Perona, P., & Meister, M. (2021). Mice in a labyrinth show rapid learning, sudden insight, and efficient exploration. *Elife*, *10*. https://doi.org/10.7554/eLife.66175

Sato, N., Fujishita, C., & Yamagishi, A. (2018). To take or not to take the shortcut: Flexible spatial behaviour of rats based on cognitive map in a lattice maze. *Behav Processes*, *151*, 39-43. https://doi.org/10.1016/j.beproc.2018.03.010

Schmitzer-Torbert, N., & Redish, A. D. (2002). Development of path stereotypy in a single day in rats on a multiple-T maze. *Arch Ital Biol*, *140*(4), 295-301. https://www.ncbi.nlm.nih.gov/pubmed/12228982

Small, W. S. (1901). Experimental Study of the Mental Processes of the Rat. II. *The American Journal of Psychology*, *12*(2). https://doi.org/10.2307/1412534

Snygg, D. (1935). Mazes in Which Rats Take the Longer Path to Food. *The Journal of Psychology*, *1*(1), 153-166. https://doi.org/10.1080/00223980.1935.9917250

Steele, R. J., & Morris, R. G. (1999). Delay-dependent impairment of a matching-to-place task with chronic and intrahippocampal infusion of the NMDA-antagonist D-AP5. *Hippocampus*, *9*(2), 118-136. https://doi.org/10.1002/(SICI)1098-1063(1999)9:2<118::AID-HIPO4>3.0.CO;2-8

Stone, C. P. (1928). The Reliability of Rat Learning Scores Obtained from a Modified Carr Maze. *The Pedagogical Seminary and Journal of Genetic Psychology*, *35*(4), 507-521. https://doi.org/10.1080/08856559.1928.10532169

Stone, C. P., & Nyswander, D. B. (1927). The Reliability of Rat Learning Scores from the Multiple-T Maze as Determined by Four Different Methods. *The Pedagogical Seminary and Journal of Genetic Psychology*, *34*(4), 497-524. https://doi.org/10.1080/08856559.1927.10532397

Tachiki, Y., Suzuki, Y., 2nd, Kurahashi, M., Oki, K., Mavuk, O., Nakagawa, T., Ishihara, S., Gyoten, Y., Yamamoto, A., & Imayoshi, I. (2023). Scale Space Calibrates Present and Subsequent Spatial Learning in Barnes Maze in Mice. *eNeuro*, *10*(6). https://doi.org/10.1523/ENEURO.0505-22.2023

Tanila, H., Ku, S., Kloosterman, F., & Wilson, M. A. (2018). Characteristics of CA1 place fields in a complex maze with multiple choice points. *Hippocampus*, *28*(2), 81-96. https://doi.org/10.1002/hipo.22810

Tolman, E. C. (1925). Purpose and cognition: the determiners of animal learning. *Psychological Review*, *32*(4), 285-297. https://doi.org/10.1037/h0072784

Tolman, E. C., & Honzik, C. H. (1930a). Degrees of hunger, reward and non-reward, and maze learning in rats. *University of California Publications in Psychology*.

Tolman, E. C., & Honzik, C. H. (1930b). Introduction and removal of reward, and maze performance in rats. *University of California Publications in Psychology*.

Tolman, E. C., Ritchie, B. F., & Kalish, D. (1946). Studies in spatial learning: Orientation and the short-cut. *J Exp Psychol*, *36*(1), 13-24. https://doi.org/10.1037/h0053944

Tolman, E. C., Tryon, R. C., & Jeffress, L. A. (1929). A self-recording maze with an automatic delivery table.

Trueblood, C. K. (1929). A Tunnel Maze. *The Pedagogical Seminary and Journal of Genetic Psychology*, *36*(4), 581-583. https://doi.org/10.1080/08856559.1929.10532215

Vincent, S. B. (1915). The white rat and the maze problem: The introduction of a visual control. *Journal of Animal Behavior*, *5*(1), 1-24. https://doi.org/10.1037/h0072410

Walton, A. (1930). Visual cues in maze running by the albino rat. *The Pedagogical Seminary and Journal of Genetic Psychology*, *38*(1-4), 50-77.

Warden, C. J. (1929a). A standard unit animal maze for general laboratory use. *The Pedagogical Seminary and Journal of Genetic Psychology*, *36*(1), 174-176.

Warden, C. J. (1929b). A symmetrical linear maze for use in the analysis of animal serial learning. *The Pedagogical Seminary and Journal of Genetic Psychology*, *36*(1), 177-178.

Ware, R. (1968). Development of differential reinforcing values of sexual responses in the male albino rat. *J Comp Physiol Psychol*, *65*(3), 461-465. https://doi.org/10.1037/h0025837

Watson, J. B. (1907). Kinæsthetic and organic sensations: Their role in the reactions of the white rat to the maze. *The Psychological Review: Monograph Supplements*, *8*(2), i-101. https://doi.org/10.1037/h0093040

Watson, J. B. (1914). A circular maze with camera lucida attachment. *Journal of Animal Behavior*, *4*(1), 56-59. https://doi.org/10.1037/h0072544

Wilson, J. J., Harding, E., Fortier, M., James, B., Donnett, M., Kerslake, A., O'Leary, A., Zhang, N., & Jeffery, K. (2015). Spatial learning by mice in three dimensions. *Behav Brain Res*, *289*, 125-132. https://doi.org/10.1016/j.bbr.2015.04.035

Wood, R. A., Bauza, M., Krupic, J., Burton, S., Delekate, A., Chan, D., & O'Keefe, J. (2018). The honeycomb maze provides a novel test to study hippocampal-dependent spatial navigation. *Nature*, *554*(7690), 102-105. https://doi.org/10.1038/nature25433

Yoder, R. M., Clark, B. J., Brown, J. E., Lamia, M. V., Valerio, S., Shinder, M. E., & Taube, J. S. (2011). Both visual and idiothetic cues contribute to head direction cell stability during navigation along complex routes. *J Neurophysiol*, *105*(6), 2989-3001. https://doi.org/10.1152/jn.01041.2010

Yoshioka, J. G. (1928). A note on a right or left going position habit with rats. *Journal of Comparative Psychology*, *8*(5), 429.
